# Unleashing the potential of combining FGFR inhibitor and immune checkpoint blockade for FGF/FGFR signaling in tumor microenvironment

**DOI:** 10.1186/s12943-023-01761-7

**Published:** 2023-03-25

**Authors:** Ruiwen Ruan, Li Li, Xuan Li, Chunye Huang, Zhanmin Zhang, Hongguang Zhong, Shaocheng Zeng, Qianqian Shi, Yang Xia, Qinru Zeng, Qin Wen, Jingyi Chen, Xiaofeng Dai, Jianping Xiong, Xiaojun Xiang, Wan Lei, Jun Deng

**Affiliations:** 1 Department of Oncology, First Affiliated Hospital of Nanchang University, Nanchang, Jiangxi Province 330006 China; 2 Jiangxi Key Laboratory for lndividualized Cancer Therapy, 17 YongwaiStreet, Donghu District, Nanchang, Jiangxi 330006 China

**Keywords:** Fibroblast growth factor receptor, Immune checkpoint blockade, Tumor microenvironment, Immunotherapy

## Abstract

**Background:**

Fibroblast growth factors (FGFs) and their receptors (FGFRs) play a crucial role in cell fate and angiogenesis, with dysregulation of the signaling axis driving tumorigenesis. Therefore, many studies have targeted FGF/FGFR signaling for cancer therapy and several FGFR inhibitors have promising results in different tumors but treatment efficiency may still be improved. The clinical use of immune checkpoint blockade (ICB) has resulted in sustained remission for patients.

**Main:**

Although there is limited data linking FGFR inhibitors and immunotherapy, preclinical research suggest that FGF/FGFR signaling is involved in regulating the tumor microenvironment (TME) including immune cells, vasculogenesis, and epithelial-mesenchymal transition (EMT). This raises the possibility that ICB in combination with FGFR-tyrosine kinase inhibitors (FGFR-TKIs) may be feasible for treatment option for patients with dysregulated FGF/FGFR signaling.

**Conclusion:**

Here, we review the role of FGF/FGFR signaling in TME regulation and the potential mechanisms of FGFR-TKI in combination with ICB. In addition, we review clinical data surrounding ICB alone or in combination with FGFR-TKI for the treatment of FGFR-dysregulated tumors, highlighting that FGFR inhibitors may sensitize the response to ICB by impacting various stages of the “cancer-immune cycle”.

## Introduction

As a member of the receptor tyrosine kinase (RTK) family, the fibroblast growth factor (FGF) receptor family promotes dimerization and activation of intracellular tyrosine residues upon ligand binding, causing recruitment of bridging proteins and docking of RAS/RAF/ mitogen-activated protein kinase (MAPK), phosphatidylinositol 3-kinase (PI3K)/ protein kinase B (AKT), phospholipase C gamma (PLCγ), and signal transducer and activator of transcription (STAT) activation of four key signaling cascades [1, 2]. Through these signaling cascades, FGF signaling can regulate cell growth, proliferation, and survival, along with other functions [3, 4]. When the highly conserved cascade becomes dysfunctional, a series of intracellular events drive tumorigenesis [5, 6], tumor cell metastasis, angiogenesis, and immune evasion [7]. The results of preclinical experiments targeting FGFR variants are encouraging, and antitumor effects have been observed in mouse models of various tumors (including breast, lung, gastric, and urothelial cancers) [7]. FGFR-tyrosine kinase inhibitors (FGFR-TKIs) are also being progressively applied in the clinic to bring effective disease control to patients [6], but in some early clinical trials only 10% of patients achieved remission [7]. This may be related to the co-expression of different FGFRs, Klothoβ expression, bypass signaling activation, and tumor heterogeneity [7, 8]. Although some benefits have been achieved in certain tumors, FGFR-TKI therapy should be further improved. In an attempt to fully and accurately elucidate the impact of FGFR signaling on tumor progression, preclinical trials pointed towards its involvement in the formation of the tumor microenvironment (TME) and further explored the rationale for the use of FGFR-TKIs in combination with immune agents. Analysis of data from early clinical trials also suggests that the combination of FGFR-TKIs and ICB could enhance therapeutic efficacy [9].

This paper reviews the regulatory role of the FGFR signaling pathway on the TME and the immune landscape. FGFR signaling can form abnormal vascular systems and promote interstitial changes but seems to be more involved in inducing the formation of an immunosuppressive microenvironment [17, 18]. This may be the result of FGFR signaling inhibiting T-cell activation and infiltration through factors such as interferon-γ (IFN-γ), granzyme B (GZMB) and chemokines, promoting macrophage M2-type transformation and recruitment and maintaining the presence of Myeloid-derived suppressor cells (MDSCs) [19–21]. However, in recent years, many studies have explored the effects of FGFR signaling on the microenvironment of different tumors in detail; some reach contradictory conclusions, which we speculate may be related to tumor heterogeneity [22–25]. Subsequently, we analyzed the impact of ICB treatment on dysregulated FGFR signaling and found inconsistent results from various studies. Preclinical studies have shown improved efficacy of ICI combined with FGFR-TKIs, possibly because FGFR-TKIs boost the cancer-immune cycle. However, more clinical trial data and stronger evidence are needed to support the use of FGFR-TKIs in combination with ICB in the clinical treatment of different tumors.

ICBs, which restore host anti-tumor and immune effects by blocking immune checkpoints (e.g., cytotoxic T-lymphocyte associated protein 4 [CTLA-4], programmed cell death protein 1 [PD-1]/programmed death-ligand 1 [PD-L1]), have achieved breakthroughs in multiple clinical practices and are gradually redefining the treatment paradigm for tumors [10, 11]. However, long-term survival data for ipilimumab (anti-CTLA-4) for melanoma suggest that only approximately 21% of patients achieve long-term (5–10 years) disease control or exhibit evidence of clinical response [12]. Others exhibited non-responsiveness (primary resistance) or early progression (acquired resistance) to immunotherapy [13]. Such differences in treatment efficacy between patients may be due to genetic heterogeneity. Mutations in certain specific loci (e.g. *EGFR* and *ALK*) are associated with innate immune resistance and can act on the TME, leading to poor treatment efficacy [14]. FGFR signaling is also involved in the regulation of the TME, and its mechanisms of action are complex and have been discussed in depth [15]. Previously, some researchers proposed a “cancer-immune cycle” theory [16]. Dendritic cells (DCs) capture and process neoantigens released by tumors and present them to T-cells. This presentation of cancer-specific antigens induces the differentiation and activation of naïve T-cells into effector T-cells. The effector T-cells then traffic to the tumor site and infiltrate it and the surrounding tumor bed through lymph nodes and blood vessels to specifically identify and kill target cancer cells. The dead cancer cells release additional tumor-associated antigens, leading to a deeper and wider immune response. Thus, T-cells need to undergo complex signal transduction to exert anti-tumor effects. FGFR signaling mediates several of these processes, which may be a potential mechanism for the anti-FGFR treatment of sensitized ICB.

## FGF/FGFR signaling oncogenic dysregulation and treatment

**Fig. 1:**
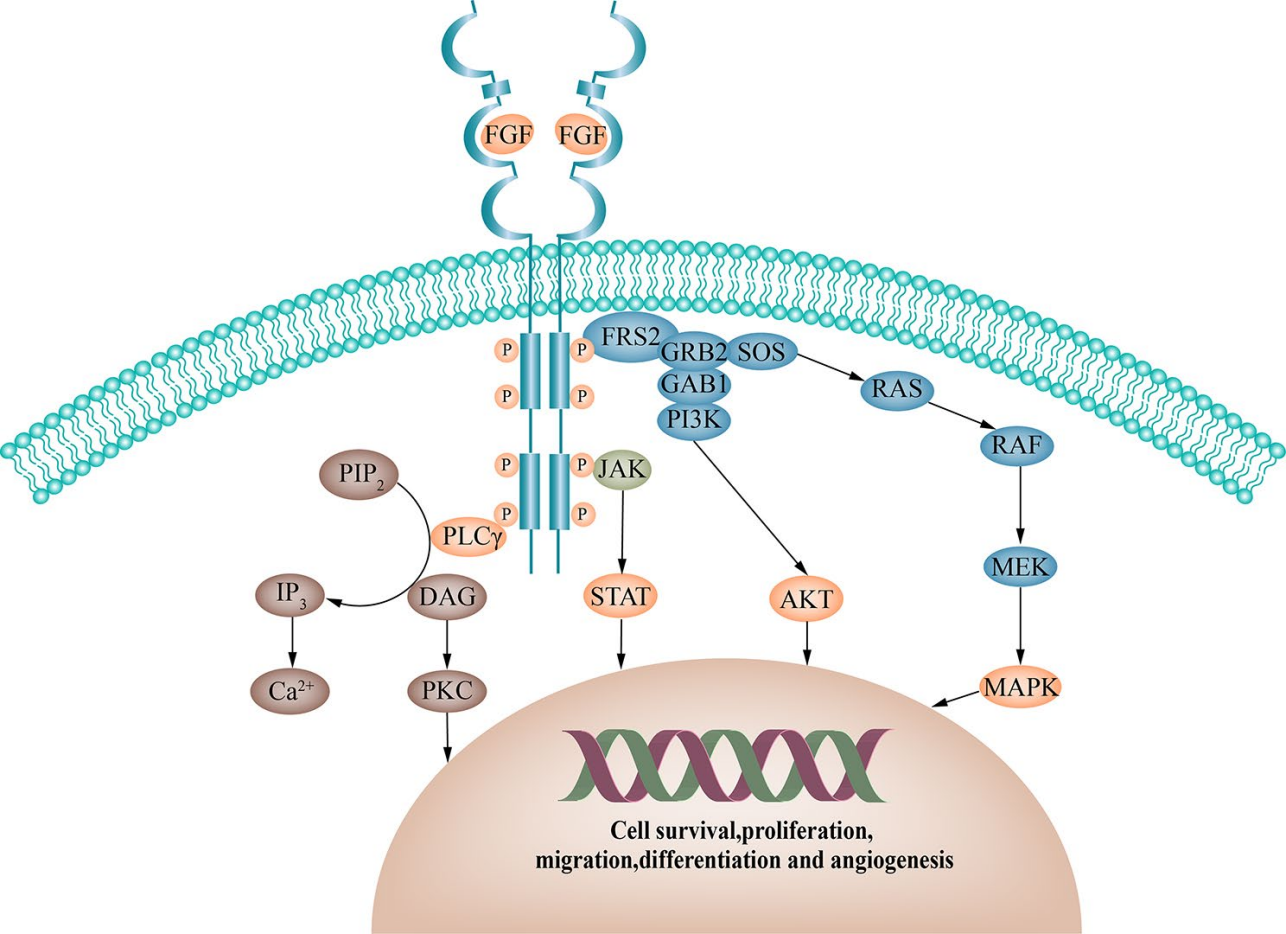
**FGFR signaling network.** Upon ligand-receptor binding, activation regions are mutually transphosphorylated, leading to the docking of junctional proteins and activation of four key downstream pathways: RAS/RAF/MAPK, PI3K/AKT, PLCγ, and STAT (orange). PIP_2_, phosphatidylinositol-4,5-bisphosphate; IP_3_, inositol triphosphate; PLCγ, phospholipase C gamma; DAG, diacylglycerol; PKC, protein kinase C; FRS2α, FGFR substrate 2α; GRB2, growth factor receptor-bound 2; GAB1, GRB2-associated binder-1; SoS, son of sevenless; P, phosphorylation; JAK, Janus kinase; PI3K, phosphatidylinositol 3-kinase; STAT, signal transducer and activator of transcription; AKT, protein kinase B; MAPK, mitogen-activated protein kinase

FGF was originally extracted from the brain and pituitary gland as a mitogen for fibroblasts, and the 22 identified FGFs are divided into two major groups according to their function; secreted signaling proteins that signal to RTK(FGF1-10,16–23), and intracellular non-signaling voltage-gated sodium channel proteins(FGF11-14) [1, 26]. as FGFR ligands, secretory signaling proteins are further subdivided into five paracrine subfamilies (heparin/ heparan sulfate (HS) for cofactor-mediated receptor binding) and one endocrine subfamily (Klotho family of cofactor-mediated receptor binding proteins), in which heparan-sulfate proteoglycans(HPSGs) and Klothoβ, cofactors in the FGFR signaling pathway, play a role in enhancing the efficiency of FGF/FGFR binding [2, 27]. The receptors associated with FGFs include the highly conserved RTK family consisting of FGFR1-4, whose internal structure includes three extracellular immunoglobulin folds, a transmembrane region and an intracellular kinase domain [3, 28]. In addition, there exists a subtype of FGFRL1 (also known as FGFR5) that does not carry an intracellular kinase structural domain [29, 30]. FGFs bind to FGFRs which then dimerize to activate the tyrosine activation domain. This activates four key downstream signaling cascades, namely PI3K/AKT, RAS/MAPK, JAK/STAT and PLCγ pathways, either directly or indirectly dependent on FGFR substrate 2 (FRS2) [3], regulating cell growth, metastasis, immunomodulation, and angiogenesis, among other effects [6, 7] (Fig. 1). Depending on the cellular environment, FGFRs also activate the Jun N-terminal kinase pathway and ribosomal protein S6 kinase 2 signaling (RSK2) [3, 31].

FGFR is highly conserved in organisms and regulates key cellular behaviors such as proliferation, differentiation, and survival [3, 5]. Thus, stable FGFR signaling is important for stable cellular functioning, and multiple intracellular and extracellular signals are cross-linked to maintain this homeostasis. Positive signals downstream of the FGFR pathway, such as FRS2α, can be fine-tuned by the negative feedback regulation of certain regulators [32, 33]. In addition, cell adhesion molecules (CAMs), G protein-coupled receptors (GPCRs), and other members of the RTK family can interact with FGFR signaling to regulate cell fate [2, 34–39]. FGF-FGFR signaling is activated by L1CAM, fibrillin leucine-rich transmembrane protein 3 (FLRT3), and FGF binding protein (FGFBP) [27, 40–42] while the Sprouty protein family, germin, MAPK phosphatase 3 (MKP3), similar expression to FGF(SEF) and other proteins are involved in inhibiting it [43]. Thus, FGFR is highly conserved in organisms and plays key role in driving cell proliferation, migration and survival [43].

Under ligand-dependent or ligand-independent conditions, the function of the FGF/FGFR signaling axis is hijacked by tumor cells, leading to oncogenic behavior [3, 6]. The mechanisms of FGF/FGFR oncogenic dysregulation include genetic alterations (gene amplification, activating mutations, oncogenic fusions), autocrine and paracrine signaling (e.g., FGF signaling), angiogenesis, and epithelial-mesenchymal transition [3, 5, 6]. These dysregulations promote the occurrence of proliferation, survival, migration, invasion, angiogenesis and other oncogenic behaviors, thus promoting tumorigenesis [3, 6].

Analysis of the molecular profiles of clinical patients revealed a 7.1% incidence of *FGFR* variants in malignant tumors [44]. The mutant forms of *FGFR* may differ in different tumors: *FGFR1* gene amplification is associated with squamous non-small cell lung cancer and breast cancer; *FGFR2* mutation is linked to endometrial cancer and gastric cancer; *FGFR2* gene fusion implicated in hepatocellular carcinoma(HCC); *FGFR3* fusion seen in myeloma; *FGFR3* mutation is associated with identified in bladder cancer; and *FGFR4* amplification/mutation has been identified in patients with rhabdomyosarcoma [44].

In the era of precision medicine, it is of particular significance to explore the relationship between FGFR dysregulation and tumor treatment. More recently, preclinical trials targeting FGFR have yielded promising results. Experiments showed that liver metastases significantly regressed after blocking FGF2 signaling. The liver tumor load of FGF2-deficient mice injected with colon, pancreatic, and lung cancer cell lines were all significantly reduced compared with that of wild-type (WT) mice [45]. However, clinical results are in consistent. In early clinical trials in HCC, the overall effectiveness of FGFR-TKI in tumor treatment was only 7-17%, and most patients exhibited only partial responses [46]. The results of several clinical studies of FGFR inhibitors in the treatment of FGFR2 fusion-positive intrahepatic cholangiocarcinoma (ICC) patients also showed objective remission rates of < 50% [47]. In addition, the *FGF/FGFR* genes were only genetically altered in 13–21% of ICC and < 20% of HCC cases [8, 47]. This low efficiency of FGFR-TKI may be influenced by co-expression of different FGFRFGFRs, expression of Klothoβ, activation of bypass signaling, and tumor heterogeneity [7]. The literature indicates that inactivation of FGFR4 alone is not sufficient to inhibit the proliferation of FGF19-positive HCC cells and suggests that Klothoβ, FGFR3, and FGFR4 together mediate the survival function of FGF19 [8].

To achieve individualized treatment and enhance therapeutic efficiency, studies in recent years have continued to explore the relationship between FGFR dysregulation and tumorigenesis and treatment. Many studies have focused on the involvement of oncogenic *FGF/FGFR* signaling in the regulation of the TME and contributes to the progression of certain tumors [7, 15, 48–51]. Furthermore, a correlation between FGF/FGFR dysregulation and immunotherapy may be likely. The rationale for FGFR-TKI coupled with ICI therapy is increasingly being explored [9, 52].

## FGF/FGFR signaling and TME

**Fig. 2:**
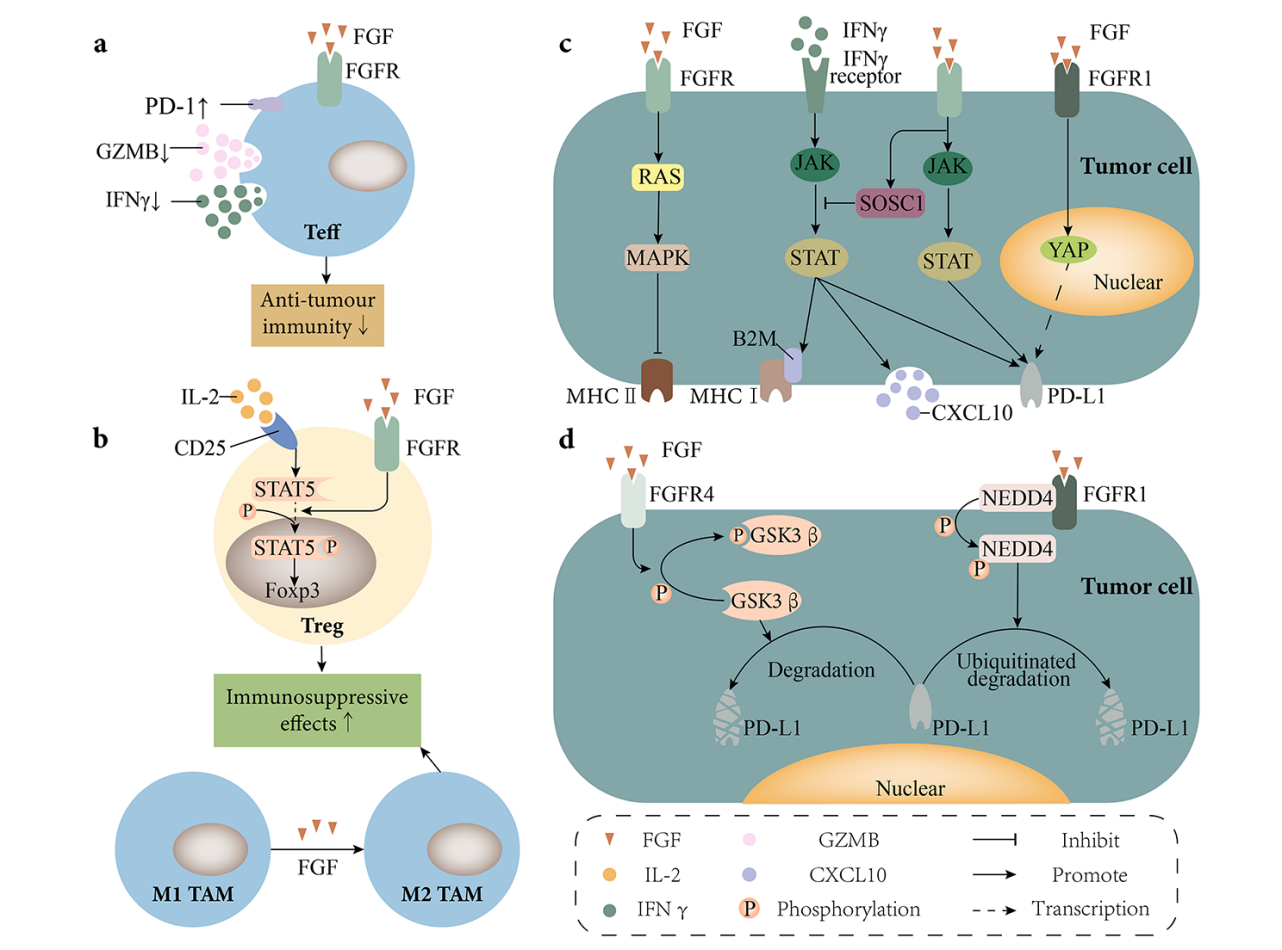
**Effects of FGFR signaling on the TME**. **a.**| FGF/FGFR promotes PD-1 expression and reduces IFNy and GZMB secretion by effector T cells, resulting in a decrease in anti-tumor immunity of T-cells. **b.**| FGF/FGFR promotes Treg cell survival by assisting IL-2-mediated STAT5 phosphorylation. FGF also promotes the M2-type polarization of TAMs. These functions of FGF/FGFR enhance immunosuppressive effects. **c.**| FGF/FGFR signaling directly inhibits MHC II expression via the RAS/MAPK pathway. At the same time, FGF/FGFR signaling also inhibited interferon-mediated expression of MHC I and PD-L1 and secretion of CXCL10 via SOSC1. In addition, FGF/FGFR signaling promotes PD-L1 expression through the JAK/STAT pathway and initiates PD-L1 transcription through the upregulation of YAP. **d.**| On the one hand, FGFR4 reduces PD-L1 degradation by promoting GSK3β phosphorylation at the Ser 9 site. On the other hand, FGFR1 promotes the degradation of ubiquitinated PD-L1 by promoting NEDD4 phosphorylation. IFN-y, interferon-γ; GZMB, granzyme B; TAM, tumor-associated macrophage; MHC, major histocompatibility complex; PD-L1, SOSC1, suppressor of cytokine signaling 1; GSK3β, glycogen synthase kinase 3 beta

**Fig. 3:**
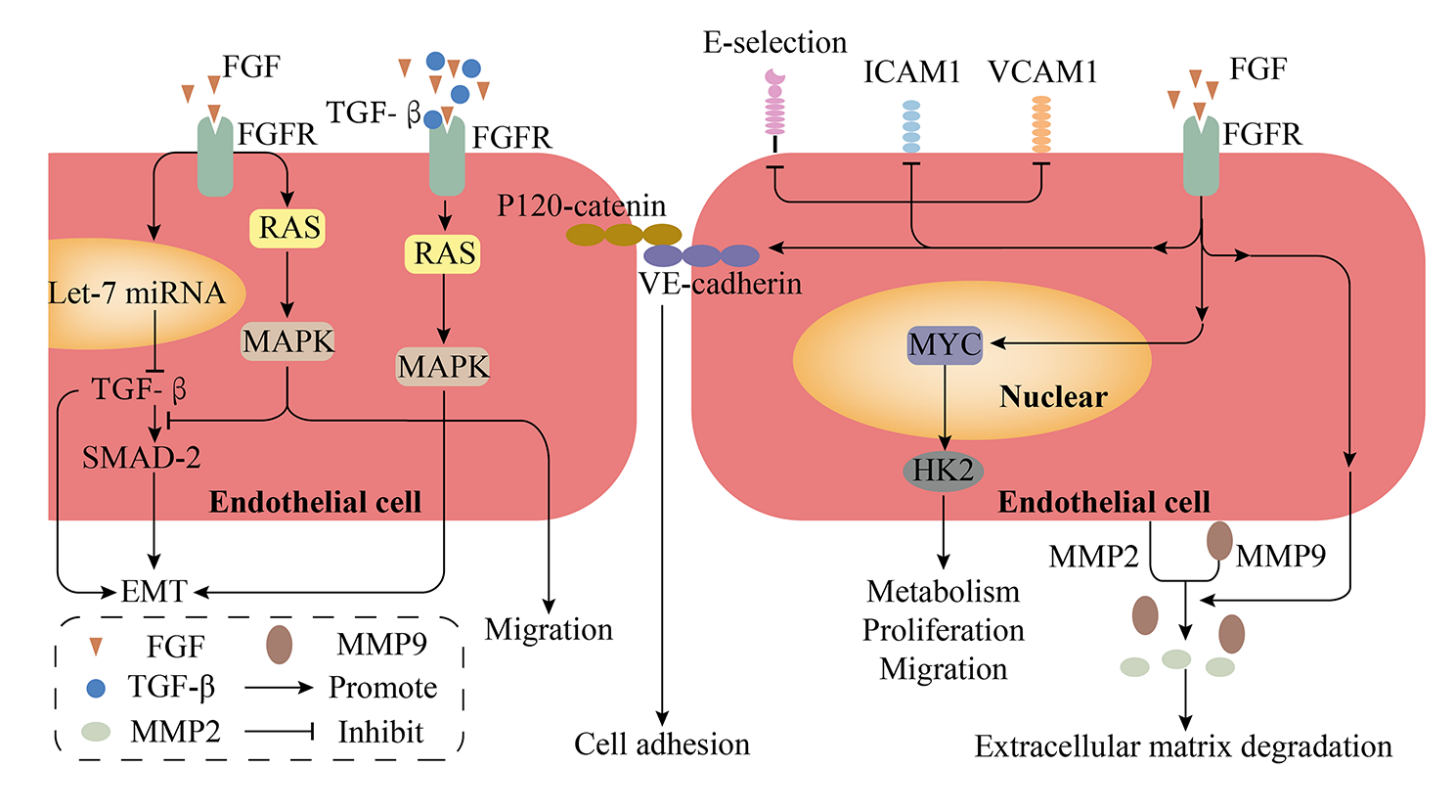
**Effects of FGFR signaling on the vascular system.** On the one hand, TGF-β facilitates the heterodimeric conversion of FGFR to promote EMT. On the other hand, FGF/FGFR signaling blocks TGF-β activation via let-7 miRNA and Smad2 activation via RAS/MAPK signaling, thus preventing EMT in endothelial cells. FGF promotes endothelial cell adhesion and tight junctions through the coupling of p120-cadherin and VE-cadherin and stimulates matrix metalloproteinase 2 (MMP2) and MMP9 shedding from the cell surface, leading to ECM degradation and promoting angiogenesis. FGF/FGFR also controls the expression of the glycolytic enzyme hexokinase 2 (HK2) via c-MYC, which assists in the metabolism, proliferation and migration of vascular endothelial cells. FGF is involved in the inhibition of expression and secretion of endothelial VCAM1, E-selectin, and ICAM1, which in turn impairs T-cell homing and recruitment. TGF-β, transforming growth factor beta; EMT, epithelial-mesenchymal transition; VCAM1, vascular cell adhesion molecule 1; ICAM1, intercellular cell adhesion molecule 1; ECM, extracellular matrix

During tumor development, individual cancer cells do not act in isolation, but together with immune cells, stromal cells, the extracellular matrix (ECM) and the vascular–lymph node network, forming a complex working network. Together, they constitute the TME, which plays an important role in the dynamic regulation of tumor progression and influences therapeutic outcomes. Recently, increasing research has also focused on treating malignancies by targeting the TME, including ways to ablate the abnormal vascular system, re-educate stromal cells, inhibit tumor-associated macrophage(TAM) recruitment, and restore T-cell depletion [53]. Previous studies have pointed out that mutations in tumor-driven pathways can lead to the activation of immune resistance or dysregulation of immune responses by acting on the TME, thereby promoting immune escape by tumors. Surprisingly, there is growing evidence that activation of the FGF/FGFR pathway is related to the TME (Figs. 2 and 3). In this section, we discuss the role of FGF/FGFR signals in the TME, and the effects of these signals on different cells in the TME are summarized in Table 1.

**Table 1:** Effects of FGF/FGFR signaling on cells in tumor microenvironment

Cell of TME	Trigger signal	Mechanism	Effect
T-cell	bFGF + VEGFA [22], FGFR [23]	The expression of PD-1, CTLA-4 and TIM-3 in T-cells was up-regulated	T-cell Exhaustion↑
	bFGF + VEGFA [22], VEGFR + FGFR [62], FGFR [63], FGFR1 [59], FGFR3 [20, 55], FGFR4 [24]	Inhibit the production of IFN-γ and GZMB	T-cell infiltration↓
	FGFR3 [66]	Upward PPARG signal	
	FGFR [115, 121]	Indirectly inhibited the recruitment of CD8 + T- cells via CXCL16	
	FGFR1 [54, 71, 72]	Promote IL-2 production by activating NF-κB	T-cell infiltration↑
Tumor-cell	FGFR [23], FGFR4 [24], FGFR2 [89],FGFR1 [88]	The expression of PD-L1 in tumors was up-regulated	Immune escape↑
	FGFR [63],FGFR1 [59],	Inhibit the expression of MHC I and MHC II molecules in tumor cells	
	FGFR3 [25]	Promote PD-L1 degradation in tumor cells	Immune escape↓
Macrophage	FGFR1 [19]	Promote macrophage recruitment through induction of CX3CL1 expression	Immunosuppression↑
	FGF2 [45]	Promote M2-type polarization of macrophages	
Treg-cell	FGFR4 [24]	Promote the differentiation and survival of Treg cells by regulating IL-2	
Epithelial-cell	FGF2-FGFR1/2IIIc [86, 131–133]	The FGF2-FGFR1/2IIIc signaling axis promotes cellular EMT	EMT↑
	FGF2 [134], FGF [135]	FGF signal inhibits EMT by blocking TGF-β	EMT↓
Endothelial-cell	FGF2-FGFR [17]	Enhance endothelial cell chemotaxis by regulating MAPK signaling	Endothelial migration and generation ↑
	FGF [155]	Promote extracellular matrix degradation by stimulating the shedding of MMP2 and MMP9	
	FGF2/FGFR1 [18]	Control endothelial cell energy metabolism by inducing HK2 expression	
	FGF [152]	Involved in inhibiting the expression of endothelial cell adhesion molecules	T-cell homing and recruitment↓

### T-lymphocyte infiltration

Preclinical experiments involving a variety of tumors point to the association of dysregulated FGF/FGFR signaling with the non-T-cell inflammatory phenotype of tumors. This is associated not only with T-cell exhaustion due to increased PD-1 expression, but also with decreased clonality and infiltration. FGFR4 expression is negatively correlated with the number of infiltrating lymphocytes in gastric malignancies [54]. *FGFR3*-mutant urothelial carcinomas have also been noted to possess an immune microenvironment background with increased T-cell exhaustion [55]. Experiments have also shown that bFGF and VEGFA signaling upregulates PD-1, CTLA-4 and TIM-3 expression in T-cells, leading to T-cell exhaustion [22]. Genetically engineered mouse models (GEMMs) of lung cancer and immunohistochemistry of human lung cancer tissues have demonstrated that the TME of *FGFR*-mutant tumors is characterized by low T-cell infiltration and high PD-L1 expression in tumors and immune cells [23]. Consistently, elevated CD4^+^ and CD8^+^ T-cells expression was experimentally detected in FGF2 signaling-deficient mice [45].

The mechanisms of action underlying the T-cell depletion phenotype have been extensively studied, constructing complex regulatory networks that emphasize the role of the IFN-γ and peroxisome proliferator-activated receptor-gamma(PPARG) pathways and Nuclear factor-kappa B(NF-kB) signaling [21, 56, 57]. At the same time, the FGFR pathway has been noted to lead to a non-T-cell infiltrating phenotype, possibly through these networks.

FGFR alterations may promote a non-T-cell infiltrative phenotype by inhibiting the production of IFN-γ and GZMB (Fig. 2a). IFN-γ is an important intermediate factor in tumor immunity that exerts its antitumor effects mainly by promoting T-cell differentiation and immune cell recruitment [58]. Inhibition of FGFR signaling in head and neck squamous cell carcinoma(HNSCC) can upregulate IFN-γ [59]. Analysis of data sets from clinical trials revealed that reduced T-cell infiltration is associated with impaired FGFR expression and IFN-γ signaling [20, 60, 61]. Combined stimulation of VEGFA and bFGF inhibits IFN-γ and GZMB secretion by HCC patient-derived T-cells [22]. The use of anti-VEGFR and anti-FGFR monoclonal antibodies increases the percentage of IFN-γ and GZMB secreted by activated CD8^+^ T-cells [62]. Furthermore, inhibition of FGFR attenuates PD-L1 expression mediated through IFN-γ, as inhibition of FGFR in the absence of IFN-γ has no regulatory effect on PD-L1 expression [24]. Meanwhile, FGFR3 knockdown results in significant upregulation of the IFN-responsive genes *BST2* and *IRF9* [20, 55]. A study on renal cancer further showed that activated FGFR signaling inhibits the IFN-γ-mediated JAK/STAT signaling pathway and consequently reduces the expression of β2-microglobulin(β2M), PD-L1, and CXCL10 [63](Fig. 2c). These proteins are directly associated with activation of the antitumor immune system.

In addition, FGFR alterations may also promote an inflammatory phenotype through the upregulation of PPARG signaling. Numerous studies have confirmed that PPARG signaling is associated with non-T-cell infiltration phenotypes of tumors [55, 64, 65], while the β-catenin, PPARG and FGFR3 pathways are simultaneously activated in non-T-cell inflammatory tumors [21]. Genomic analysis of *FGFR3-*mutant tumors compared with WT tumors found that upregulated *PPARG* gene signaling is significantly associated with *FGFR3* mutations [66]. At the same time, PPARG signaling leads to an anti-inflammatory environment through the inhibition of NF-κB [67]. Predictably, *FGFR* alterations may potentially inhibit NF-κB and reduce T-cell infiltration through PPARG pathway activation, but this remains to be corroborated by further studies.

Paradoxically, FGF/FGFR signaling dysregulation in specific tumors may instead lead to T-lymphocyte infiltration. Earlier studies pointed out that chronic immune injury (e.g. rheumatoid arthritis) drives some CD4^+^ T-cells to express FGFR1 [68–70]. This FGFR signaling promotes interleukin (IL)-2 production by degrading IκBα and activating NF-κB, which promotes T-lymphocyte proliferation by activating RAS and PI3K with the help of SNT-like proteins [71, 72]. Bioinformatic analysis of tissues from gastric cancer patients also revealed that the degree of infiltration of CD8^+^ and CD4^+^ T-cells, macrophages and DCs was positively correlated with the expression of FGFR1 in tumor cells [54]. This suggests that the mechanisms by which different FGFR activating alterations act in tumors may be different and regulated by different factors and require more detailed and comprehensive studies for elucidation.

### Major histocompatibility complex (MHC) expression

The MHC system shapes all the components of the presenting peptide and subsequent immune responses [73]. MHC molecules bind to antigenic peptides through their peptide-binding grooves and present them on the cell surface for recognition by T-cells, a process that helps stimulate subsequent anti-tumor responses [74]. However, tumors will act on MHC molecules to evade immune surveillance through several mechanisms, chief among which is altered MHC expression. Of note, FGFR signaling downregulates MHC expression(Fig. 2c). Early experiments have shown that IFN-γ plays a role in inducing the expression of MHC Class II molecules [75, 76]. In the absence of IFN-γ, upregulation of human leukocyte antigen(HLA)-DR and expression of MHC Class II transactivator (CIITA) in HNSCC are still observed after the use of FGFR inhibitors [59]. In addition, Toll-like receptor 2 can inhibit CIITA expression by inducing MAPK signaling [77]. Notably, the MAPK signaling pathway is a common pathway downstream of multiple proteins such as FGFR and EGFR [5, 78]. Combining these results, we speculate that FGFR may be linked to MHC via the MAPK pathway. Recently, a study on HNSCC demonstrated through in vivo and in vitro assays that inhibition of MAPK/EPK signaling promotes CIITA expression while that of STAT3 or PI3K does not affect HLA expression in tumors [59]. Meanwhile, other experiments showed that FGFR signaling leads to a decrease in β2M expression by inhibiting the IFN-γ-mediated JAK/STAT signaling pathway [63]. β2M is an important subunit of MHC Class I molecules and is involved in mediating its antigen presentation [79]. Thus, FGFR signaling indirectly inhibits the expression of MHC Class I molecules by tumor cells through inhibition of the IFN-γ-mediated JAK/STAT signaling pathway and inhibits the expression of MHC Class II molecules in tumor cells on the other, thereby reducing T-cell infiltration and promoting immune escape.

### PD-1/PD-L1 regulation

Macrophages, T-lymphocytes, natural killer (NK) cells and dendritic cells (DC) in the TME together construct an antitumor innate immune defense [80]. In addition to this, the adaptive immune response is also critical in the TME and involves the activation of T-lymphocytes and B-lymphocytes, which are capable of recognizing and responding to specific antigens. Together, the innate and adaptive immune responses work to provide a multi-layered defense against the tumor threat. DCs, CD4 + T helper (Th) cells, and CD8 + cytotoxic T-lymphocytes (CTL) play a central role in antitumor immunity and are significantly associated with tumor prognosis [81]. T-cells rely on the combined involvement of antigenic peptide-MHC complexes, co-receptors, and co-stimulatory signals to achieve effective activation [82–84]. Expression of multiple co-inhibitory molecules by T-cells results in a significant reduction in T-cell proliferation and effector function [83]. For example, PD-1 can interfere with T-cell antigen receptor-mediated signaling and thereby inhibiting T-cell responses [85]. Such depleted T-cells expressing co-repressor molecules exhibit limited anti-tumor responses.

Consistent with this immune exhaustion, FGFR signaling can lead to immune resistance through the upregulation of PD-1/PD-L1 [86, 87] (Fig. 2a, c, d). A mouse model of HCC demonstrated that VEGFR and FGFR signaling inhibit the secretion of IFN-γ and GZMB from T-cells, significantly upregulating PD-1 expression in T-cells and PD-L1 in tumor cells [22, 24]. More importantly, such experiments further suggested that PD-L1 expression may be dependent on FGFR4-mediated phosphorylation of glycogen synthase kinase 3 beta (GSK3β) at Ser9, which affects the stability of PD-L1 rather than its transcription [24]. In addition, in lung squamous cell carcinoma, FGFR1 also initiates PD-L1 transcription through YAP upregulation [88]. In vitro colon cancer assays demonstrated that FGFR2 signaling promotes PD-L1 expression through the JAK/STAT signaling pathway and is not affected by mammalian target of rapamycin (mTOR) or MAPK signaling [89]. In contrast, in vitro trials in lung adenocarcinoma cases found no reduction in PD-L1 expression by tumor cells after blockade using inhibitors of mTOR, MEK1/2, JAK1/2, or STAT3/5 alone, suggesting that achieving complete regulation of PD-L1 expression may require simultaneous blockade of several FGFR downstream pathways [23].

However, not all FGFR signaling in all tissues promotes PD-L1/PD-1 upregulation. Positive PD-L1 expression by tumor cells was observed in only one of 58 FGFR2 fusion-positive ICC patients, but was detected in eight of the FGFR2 fusion-negative specimens [47]. Genetic clustering analysis of 489 ICC cases noted that no significant PD-1/PD-L1 alterations were seen in FGFR expression clusters [90]. These studies suggest that FGFR signaling in ICC may not be significantly associated with PD-1 expression. Moreover, in bladder urothelial carcinoma with FGFR-activating mutations, FGFR3 promotes PD-L1 degradation via NEDD4 [25] (Fig. 2d). Analysis of patients with bladder urothelial carcinoma in some early clinical trials and real-world studies also revealed that lower PD-L1 expression is observed in *FGFR3*-mutated tumors [91–94].

Thus, the regulation of PD-L1/PD-1 expression by FGFR signaling is a complex process with paradoxical outcomes that may depend on the histological context.

### Regulation of immunosuppressive cell infiltration and macrophage polarization

Tumor development is also a process accompanied by the recruitment and activation of immunosuppressive cells (including macrophages, MDSCs, regulatory T [Treg] cells). Cells such as M2-TAMs, Treg cells, and MDSCs in the TME are an important part of tumor evasion from immune cell surveillance and destruction [80]. Macrophages exhibit significant plasticity throughout tumorigenesis [95]. Macrophages in the earliest stages of tumorigenesis are involved in tumor clearance in concert with T-cells [95]. Subsequently, stimulated by various cytokines in the TME, TAMs polarize into the M2 type and promote tumor growth through mechanisms that regulate angiogenesis, tumor cell proliferation, metastatic potential, chemoresistance, and immune escape [45, 96–99]. As a group of immature myeloid cells capable of suppressing immune responses, MDSCs inhibit the immune response mainly by acting on the tumor immune environment (including activation of M2-TAM and Treg cells and suppression of CD8^+^ T-cells and NK cells), blocking lymphocyte homing, and promoting epithelial–mesenchymal transition (EMT) and angiogenesis [100–105]. Th2 subtype CD4^+^ T-cells and Treg cells also have anti-tumor immune responses that suppress CTL and NK cells [98, 106–108].

Activation of FGFR promotes leukocyte recruitment, with macrophages being the most dominant T-cell type [109, 110]. Increased infiltration by TAMs and Treg cells is observed in FGFR-expressing lung malignancies [23]. Analysis of a dataset of triple-negative breast cancers revealed that tumors with high FGFR expression have decreased infiltration of CD8^+^ T-cells and M1 macrophages but increased infiltration of M2 macrophages [111]. Relatedly, activated FGFR1 signaling promotes macrophage recruitment through NF-κB signaling-induced chemokine CX3CL1 expression [19]. However, CX3CL1 can also be involved in the recruitment of CD8^+^ T-cells, NK cells, and DCs [112]. This suggests that macrophage recruitment may not be the only role of CX3CL1.

In addition, FGF/FGFR signaling was involved in regulating the M2 polarization of macrophages and the survival of Treg cells (Fig. 2b). A previous study detected increased expression of FGFR1/2 in bone marrow-derived macrophages (BMDMs) after co-culturing with tumor cells [45]. Further experiments demonstrated that FGF2-deficient BMDMs had higher levels of pro-inflammatory cytokines (e.g., CXCL1, IL-1β, IL-6, and TNF-α) and reduced expression of M2 markers Ym1 and Ym2 compared to WT BMDMs [45]. In addition, reduced FOXP3 levels were detected after treatment with lenvatinib in a murine model of HCC, suggesting that Treg cells may be activated by FGFR signaling [24]. These results further suggest that Treg infiltration is not the result of cell recruitment but rather a result of pro-cellular differentiation and survival exerted by FGFR signaling through regulation of IL-2 [24].

Although inhibition of FGFR can reduce the infiltration of MDSCs, the mechanism of FGFR signaling on MDSCs remains to be elucidated. FGFR inhibitors have been shown to decrease proliferation and lung metastasis of breast tumors and reduce infiltration of MDSCs [49, 113–115]. The use of FGFR inhibitors decreases granulocyte colony-stimulating factor(G-CSF) levels via mTOR signaling, thereby reducing the mobilization of MDSCs [116]. However, analysis of some experimental pools revealed that FGFR indirectly inhibited the expansion of MDSCs and the recruitment of CD8^+^ T cells via CXCL16. By analyzing the results of experiments in mouse breast cancer model, it was found that inhibition of FGFR led to a significant increase in CXCL16 expression and was consistent with infiltration of CD8^+^ T cells [115, 117]. The same results were also seen in the analysis of the human bladder cancer dataset (GSE52452) [115]. CXCL16 induces MDSC expression of CD38 in vitro, which regulates MDSC amplification [118–120]. On the other hand, an important study pointed out that CXCR6, the receptor for CXCL16, plays a key role in CTL-mediated tumor control, and contributes to the survival and local expansion of effector-like CTL in the TME [121]. Meanwhile, CXCL16 expression promotes the accumulation of tumor-specific CXCR6^+^CD8^+^ T cells in tumor tissues [117, 121, 122].

### Changes within the interstitial space

EMT refers to the process by which epithelial cells acquire mesenchymal features and is associated with tumorigenesis, metastasis, invasion, immune escape, and treatment resistance [123–125]. Mesenchymal breast cancer cell lines with more EMT markers weakly express MHC Class I molecules and promote the recruitment of Treg cells and M2-TAMs [126]. EMT also promotes PD-L1 expression [126–128].

As research on this continues, the potential role of the FGF/FGFR signaling axis in EMT continues is being further revealed. Transforming growth factor β(TGF-β) plays an important role in EMT and FGF/FGFR can be used as a marker of TGF-β1 signaling [129, 130], as TGF-β acts synergistically with FGF-2 to promote EMT and tumorigenesis [131]. Specifically, TGF-β induces EMT while promoting the heterodimeric conversion of FGFR2IIIb to FGFR1IIIc on normal epithelial cells, which elevates cellular sensitivity to FGF-2 [131]. Subsequently, FGF-2 promotes the formation of phosphorylated δEF1-CtBP1 complexes through the MAPK/ERK pathway to enhance EMT [131]. Following EMT, the expression levels of FGFR1IIIc and β3 integrin increase and utilize focal adhesion kinase (FAK) to aberrantly activate ERK1/2, which also enhances the response to FGF-2 [132]. This suggests that FGFR1IIIc can drive aberrant downstream signaling in concert with other effector molecules of EMT to promote tumor growth and metastasis [132]. Furthermore, the data suggest that FGFR2IIIb expression is not associated with EMT, whereas FGFR2IIIc expression and activation contribute to the response to FGF-2, triggering EMT [133]. Thus, the switch between FGFR2IIIb and FGFR2IIIc induces EMT [133]which may be due to selective splicing facilitated by the AKT3/IWS1 pathway [86]. Notably, bladder cancer expressing FGF-2 has also been identified as a tumor subtype prone to EMT, which may be related to the induction of KDM2B and EZH2 expression by FGF-2 [86]. In conclusion, FGF-2 and FGFR1/2IIIc appear to play an important role in the development of cellular EMT in the TME.

However, the findings of other studies are different. Compared with FGFR3-WT in urothelial carcinoma, FGFR3 alteration results in low expression of TGF-β and EMT signaling [20]. In both in vivo and in vitro experiments, FGF2 signaling blocks SMAD2 activation via RAS/MAPK signaling, which inhibits TGF-β signaling and further blocks EMT in lymphatic endothelial cells(LECs) [134]. In addition, FGF signaling blocks TGF-β activation by promoting let-miRNA, which impedes EMT in vascular endothelial cells [135]. Thus, the conflicting effects of FGF/FGFR signaling on EMT may be related to the crosstalk between different FGF/FGFR signals.

Cancer associated fibroblasts (CAFs) in the TME have been shown to play an important role in tumor progression and regulation of immunity, and can support tumor growth through multiple mechanisms, including ECM deposition and matrix proteases production [136], which can provide a pathway for local invasion and migration by tumor cells and act as a barrier to immune cell infiltration [137–139]. Evidence suggests that FGF contributes to CAFs activation [45, 136]. Additionally, in breast cancer dataset (GSE114727), an increased number of fibroblasts and decreased number of CD8^+^ T-cells were observed in a high FGFR1 expression group [111]. Experiments also further indicated that blocking FGFR signaling inhibits CAF proliferation and migration by downregulating the MAPK signaling pathway, which would disrupt the physical barrier established by CAF [111]. In addition, blocking FGFR prevents CAF from secreting the vascular cell adhesion molecule 1 (VCAM1), which is closely associated with tumor metastasis and angiogenesis [111]. These alterations promote T-cell infiltration. Furthermore, FGFR signaling in breast cancer induces STAT3 activation, which results in a hyaluronic acid-rich microenvironment that contributes to tumor growth and metastasis [140].

### Abnormal vascular network

The abnormal vascular system (both lymphatic and vascular networks) in the TME manifests as elevated interstitial fluid pressure (IFP) and decreased vascular supply efficiency, which affects the supply of oxygen and drugs in the blood and increases tumor aggressiveness [141, 142]. In addition, this abnormal vascular system can prevent immune cell entry through differential expression of integrins and CAMs [143]. Thus, the vascular network system plays an important role in regulating tumor progression and immunotherapeutic efficacy [53].

Tumor vasculogenesis is a complex process driven by the VEGF, PDGF, EGF, FGF, and ANG families, with VEGF-VEGFR and FGF2-FGFR1/2 signaling playing key roles by promoting endothelial cell proliferation and migration, duct formation, and protease production [18, 144–146]. Assays on murine brain capillary endothelial cells (MBEC) point to enhanced chemotaxis of MBEC by FGF2/FGFR, an effect is associated with MAPK signaling [17, 147]. Several studies have also indicated that TAMs can further influence the immune microenvironment by secreting FGF in concert with FGFR signaling to promote angiogenesis [142, 148]. In addition, FGF/FGFR signaling stimulates matrix metalloproteinase 2 (MMP2) and MMP9 shedding from the cell surface, which leads to ECM degradation, promoting angiogenesis [149, 150]. FGF is also involved in suppressing the expression of endothelial VCAM1, E-selectin, and intercellular cell adhesion molecule 1, which in turn impairs T-cell homing and recruitment [151–153].

Interestingly, FGF-FGFR1/2 signaling is noted to play a key role in injury-induced angiogenesis without affecting vascular permeability and reactivity under physiological homeostatic conditions [154]. However, research indicates that FGF signaling also plays an important role in maintaining vascular-lymphoid endothelial cell homeostasis. As described previously, FGF/FGFR blocks EMT in vascular and LECs [134, 135]. Inhibition of FGF also results in the decoupling of p120-catenin from VE-cadherin and further disrupts the subsequent adhesion and tight junctions of arterial and venous endothelial cells, which leads to the loss of endothelial cell-cell contacts [155].

Notably, one study also linked the vascular regulatory role of FGF2/FGFR1 to endocrine metabolism [18]. In FGFR1/3 double-knockout mice, significantly impaired retinal vascular growth and branching reduced anterior migration of LECs, and reduced numbers of LECs in branches and the skin were observed [18]. Importantly, these effects were not seen with FGFR3 knockout alone, and FGFR1/3 double-knockout produced similar effects to those seen with FGFR1 knockout alone [18]. This suggests that FGFR1 signaling may be a more important component of vasculogenesis. This study further found that FGF2/FGFR1 signaling strongly induced expression of the enzyme hexokinase 2 (HK2) in glycolysis, which is strongly associated with MYC signaling [18]. Thus, FGF regulates the expression of HK2 through control of c-MYC expression, thereby promoting vascular endothelial cell proliferation and migration [18] (Fig. 3).

In summary, FGF/FGFR signaling can accelerate tumor metastasis through the formation of abnormal physical and chemical barriers (Fig. 3).

## Immunological effects of FGFR-TKI

### Involvement of FGFR-TKI in tumor microenvironment regulation

Immunogenic tumor death (ICD) is the death of tumor cells accompanied by the synthesis and release of large numbers of damage-associated molecular patterns (DAMPs), which can enhance immunogenicity and induce the recruitment and activation of antigen-presenting cells, ultimately activating innate and adaptive immune responses [156–158]. Certain targeted drugs can directly kill tumor cells, further enhancing immunogenicity by driving tumor antigen re-expression and T-cell infiltration [159, 160]. EGFR-TKI has been shown to induce ICD [161]. Recent experimental studies have also suggested that FGFR-TKIs stimulate immunogenic tumor death [23, 59]. In an FGFR-insensitive KRAS-mutant lung cancer GEMM, neither the use of FGFR inhibitors alone nor in combination with anti-PD-1 monoclonal antibodies controlled tumors to improve survival in mice [23]. Further experiments also failed to observe increased tumor T-cell infiltration, decreased Treg cells, and downregulation of PD-L1 in tumor cells in KRAS-mutant GEMM [23]. In contrast, reduced tumor clonality and an increased T-cell fraction were detected in FGFR-mutant tumors treated with FGFR inhibitors compared to those subjected to anti-PD-1 treatment therapy [23]. These results indirectly suggest that treatment with FGFR inhibitors induces tumor cell death, thus further stimulating ICD and promoting immune activation. However, this still needs to be corroborated by in-depth and direct experiments.

FGFR-TKI induces normalization of the tumor vascular system, which is a prerequisite for the extravasation of immune cells and T-lymphocyte infiltration [144, 145, 162]. Anti-angiogenic therapy including anti-FGF signaling has been well-documented in numerous studies to enhance the efficacy of immunotherapy [163–166]. Anti-VEGFR and anti-FGFR treatment significantly reduce tumor microvessel density and perfusion [22, 167]. Additionally, anti-VEGFR treatment reduces IFP, enhances osmotic pressure gradients and boosts perivascular cell coverage, promoting normalization of the vascular wall [168]. Inhibition of FGF/FGFR signaling disrupts endothelial cell adhesion and tight junctions, leading to vascular system breakdown [145], causing tumor cell death and promoting immune cell infiltration. In addition, immune cell activity is regulated by microenvironmental hypoxia and low pH, and vascular normalization helps to reverse this effect [96, 151].

FGFR-TKI has been reported to be involved in regulating T-cell activation, enhancing T-cell infiltration, and decreasing PD-1/PD-L1 expression in a variety of tumors, including lung, breast, and liver cancers. In vitro trials in both lung and breast cancers indicated that FGFR-TKI enhances infiltration of CD4^+^ helper T-cells and CD8^+^ effector T-cells and inhibits the generation of Treg cells and end-depleted T-cells [23, 114, 115]. Importantly, erdafitinib (an FGFR inhibitor) does not affect the expression of single depletion marker-positive CD4^+^ and CD8^+^ T-cells in lung cancer. Targeting FGFR also reduces IFN-γ-induced PD-L1 expression [23]. In a mouse model of liver cancer, lenvatinib (a dual inhibitor of VEGFR and FGFR) reduced the expression of PD-1 on T cells, and PD-L1 on tumor and umbilical vascular endothelial cells. Lenvatinib also promotes the secretion of IFN-γ and GZMB by CD8^+^ T-cells, which contributes to effective immune activation [22, 24, 62]. Notably, after lenvatinib treatment of HCC cells, knockdown of the *FGFR4* gene, and FGF19 stimulation, the mRNA levels of *FGFR* but not *PD-L1* were affected [24]. This suggests that FGFR signaling does not affect PD-L1 expression at the gene level. Further experiments delved deeper and indicated that lenvatinib targeting of FGFR4 in liver malignancies leads to β-phosphorylation of GSK3Ser9, which contributes to the ubiquitination and degradation of PD-L1 in HCC cells [24]. However, in a mouse bladder cancer model with *FGFR3*-activating mutations, inhibition of FGFR rescinded the ubiquitination and degradation of PD-L1 by NEDD4 and significantly reduced the ratio of Ki67, TNF-α, GZMB and perforin positivity released from activated CD8^+^ T-cells, which severely inhibits the tumor-killing effect of CD8^+^ T-cells [25]. Meanwhile, CD8^+^ T-cell depletion markers, including TIM-3, LAG-3 and PD-1, were not regulated by Erdafitinib targeting [25]. These contradicting results suggest tumor heterogeneity in terms of FGFR signaling and modulation by more intrinsic molecules.

Lenvatinib rescues IFN-γ-induced T-cell sensitivity, which helps reduce the development of acquired immune resistance [24]. Nevertheless, how such effects are produced, possibly related to MHC expression, require more comprehensive studies. FGFR-TKI possibly promotes MHC expression in tumor cells, which also affects T-cell activation [59], and also modulates TAMs programming, reduces MDSCs mobilization, and inhibits Treg cell generation, which can further prevent the immune escape of tumors [113, 114]. In contrast to acting on chemokine-mediated recruitment of TAMs and survival of MDSCs, lenvatinib blocks Treg differentiation rather than acting on chemokine-induced cell recruitment, and this effect is associated with IL-2-mediated phosphorylation of STAT5 [24].

### FGFR-TKI and cancer-immune cycle theory

The tumor immune cycle theory states that the whole process of anti-cancer immune responses consists of seven steps: production and release of tumor neoantigens, capture of antigen-presenting cells, activation of effector T-cells, transport by the vascular system, tumor bed infiltration, MHC-mediated recognition of homologous peptides, and killing of tumor cells [16, 169]. Dead tumor cells again release additional tumor-associated antigens, once again promoting circulating reactivity [16]. Furthermore, the anti-tumor effects of T-cells are influenced by a combination of intrinsic tumor properties (epigenetic alterations) and extrinsic factors (e.g., TME) [13, 170]. Combined with the theory of immune circulation, the onset of T-cell antitumor action is not a single process.

**Fig. 4:**
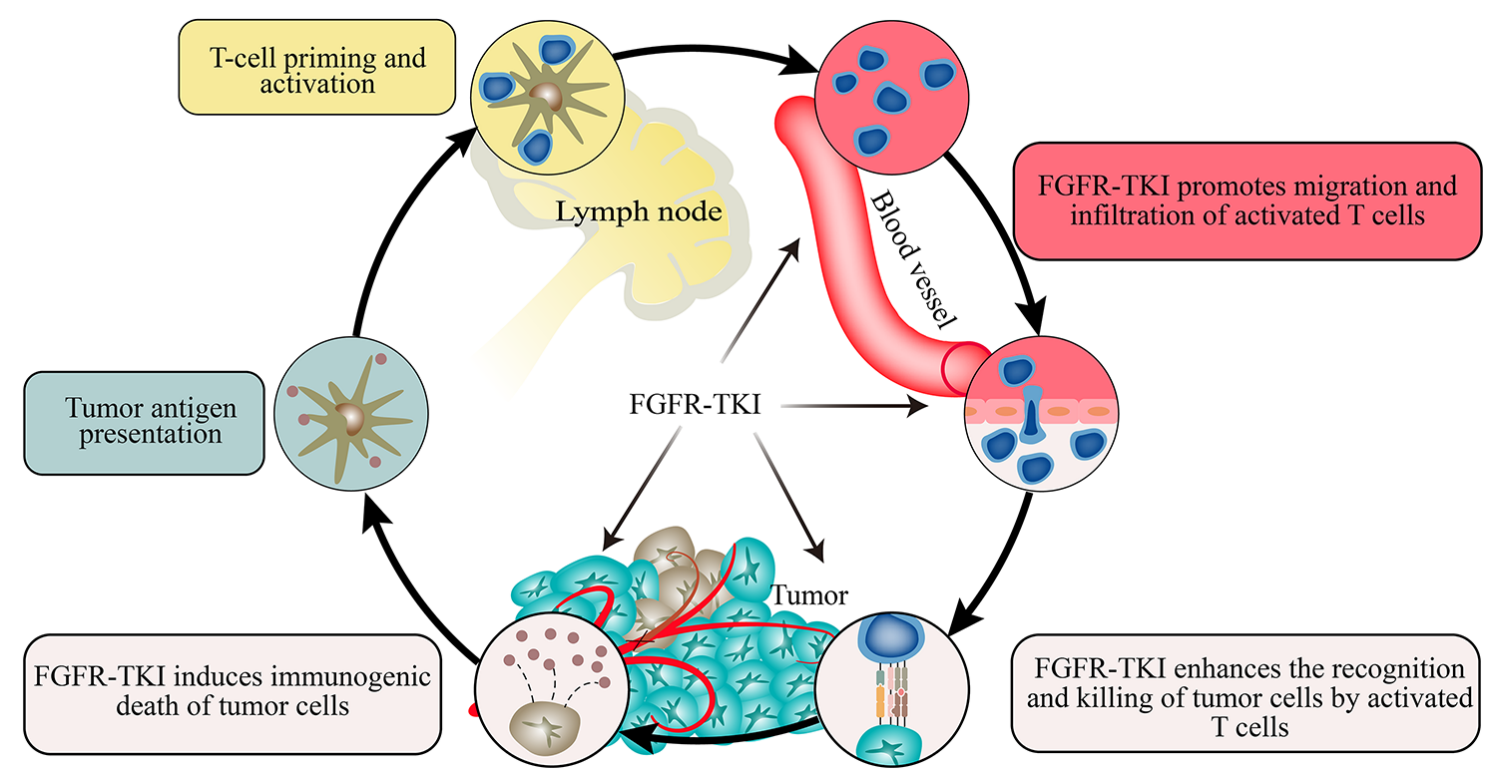
Targeting FGF/FGFR signaling to regulate the cancer-immunity cycle in multiple steps

We hypothesized that FGFR-TKI could act as an adjuvant to target T-cell activation in certain tumors in a cancer immune cycle-dependent manner (Fig. 4). First, FGFR-TKI directly causes epigenetic alterations in T-cells, promotes T-cell lineage diversification, reduces T-cell PD-1 expression, intrinsically restores T-cell anti-tumor activity and prevents active T-cell death. Second, FGFR-TKI also promotes IFN-γ and GZMB secretion and inhibits PPARG signaling, which contributes to T-cell activation and infiltration. In addition, FGFR-TKI relieves the immunosuppressive effect on T-cell activation by correcting incorrect immunosuppressive cells in the TME. FGFR-TKI remodels the tumor immune microenvironment by reducing the infiltration and survival of Treg cells and MDSCs and reversing the M2-type polarization of TAMs, which partially relieves the drug resistance response to ICB treatment via an extrinsic mechanism. In addition to this, FGFR-TKI interferes with the infiltrative migration of immune cells and promotes tumor bed infiltration of T-cells. FGFR-TKI can promote the normalization of abnormal blood vessels in tumors and exert anti-tumor effects while reducing the shunting of T-lymphocytes and drugs. They can derepress the inhibitory effect of FGFR on chemokine expression and drive T-cell homing and tumor bed infiltration. Finally, FGFR-TKI can also directly target tumor cells, resulting in increased TME tumorigenic antigen exposure, which further enhances immune responses. This tumorigenic death allows amplification of the tumor signal, contributing to activation of the immune system in vivo and promoting T-cell infiltration.

Overall, FGFR-TKI acts at various steps of the cancer-immune cycle, further assisting in the activation of T-cells.

## Combination of FGFR-TKI and immune checkpoint therapy

### Immune checkpoint therapy and oncogenic mutations

Immune checkpoints play an important role in promoting immune escape, such as CTLA-4 that blocks co-stimulatory signals thereby attenuating and terminating T-cell responses, as well as mediating the generation of immune escape [171]. By blocking immune checkpoints, ICBs can reactivate T-cell function and kill cancer cells by restoring autoimmune function [10, 11]. ICBs can reactivate T-cell function by blocking the immune checkpoint, relying on the restoration of autoimmune function to kill cancer cells. In many preclinical studies and clinical trials, ICBs, including CTLA-4 and PD-1/PD-L1 inhibitors, have achieved impressive results [171].

Although the clinical use of ICB has significantly improved the prognosis of patients with certain advanced tumors, the low positive rate of immunotherapy and the development of drug resistance are clinical problems that remain unresolved [144, 172, 173]. The anti-tumor immune response is regulated by tumor cell mutations, epigenetic alterations, and environmental factors [85]. Recent studies have pointed out that the components in TME doubly influence the efficacy of ICB [174]. In addition, the TME plays a more important role than immune checkpoints in immune escape and surveillance of tumors [151, 175, 176]. Experiments have also indicated that certain site-specific mutations may lead to innate immune resistance by acting on the TME and are associated with ICB treatment resistance. For example, patients with tumors carrying *EGFR* or *ALK* mutations have reduced levels of tumor-infiltrating lymphocytes, elevated PD-L1 expression, and benefit poorly from ICB therapy [14, 177–181]. Granulocyte-macrophage colony-stimulating factor produced by KRAS^G12D^ mutant pancreatic cancer also leads to suppression of CD8^+^ T-cells and depletion and recruitment of MDSCs, thereby reducing the efficacy of immunotherapy [182]. FGFR is also involved in the formation of TME and seems to be targeted as a potential immune resistance-related molecular site.

### Role of FGFR dysregulation in determining tumor response to PD-1/PD-L1 inhibitors

As mentioned previously, oncogenic *FGFR* mutations are closely associated with a non-inflammatory environment in certain malignancies, which may lead to poor responses to ICB treatment [9]. It has been demonstrated that overexpression of FGFR4 impairs the immunotherapeutic effect in gastric malignancies [54]. Consistently, a mathematical model of bladder cancer has shown that FGFR WT tumors are more susceptible to anti-PD-L1 therapy than *FGFR*-mutated tumors [183]. An analysis of melanoma patients treated with immunotherapy revealed relatively higher *FGFR1* mRNA expression in pretreatment tumors in non-responders compared to responders [111]. Furthermore, compared with low FGFR-expressing patients, high FGFR1-expressing patients receiving anti-PD-1 therapy had shorter overall survival(OS) and high FGFR3-expressing patients had shorter progression-free survival (PFS) [111]. Meanwhile, the results of anti-PD-L1 therapy in patients with advanced urothelial carcinoma showed that the median OS was lower in FGFR-positive patients than in FGFR-negative patients (3.1 months vs. 6.1 months; hazard ratio [HR], 1.33; 95% confidence interval[CI], 0.78–2.26, P = 0.30) [184, 185]. Patients with FGFR-positive urothelial carcinoma also had a lower response rate to PD-(L)1 therapy compared to FGFR-negative patients [186]. In addition, sequencing of samples from patients with hepatocellular carcinoma treated with immunotherapy combined with anti-angiogenic therapy revealed that FGF3/4/19 amplification may also lead to a high rate of disease progression [187].

In fact, a proportion of patients experience rapid cancer progression during immunotherapy, namely hyperprogressive disease (HPD) [188]. Patients with FGF3, FGF4 and FGF19 amplification tumors were reportedly more prone to HPD after immunotherapy [189]. A recent study has elucidated the molecular basis of HPD in ICI patients and indicated the key role of FGF-2 [190]. It unexpectedly found that patients with complete response and HPD exhibited similar levels of IFN-γ and Interferon Regulatory Factor 1(IRF1), as well as similar T-cell infiltration during immunotherapy, suggesting that patients with HPD are not exclusively associated with immune exclusion [190]. IFN-γ is known to play a dual role in antitumor immunity and immune escape [58, 191, 192]. The data also indicate that ICI can promote tumor growth in a CD8^+^ T-cell-dependent manner [190]. Thus, ICI may contribute to HPD by activating oncogenic pathways through IFN-γ signaling from T-cells [190]. Further experiments indicated that IFN-γ activated β-catenin signaling in HPD tumor cells as a result of it reducing NAD^+^ levels to enhance β-catenin acetylation [190]. Moreover, IFN-γ phosphorylates M2 pyruvate kinase(PKM2) to an inactive form [190]. PKM2 activation blocks IFN-γ-mediated β-catenin activation and subsequent HPD [190]. Thus, IFN-γ targets PKM2 to diminish sirtuin-mediated β-catenin deacetylation via NAD^+^ reduction, causing β-catenin acetylation and activation [190]. Subsequent experiments indicated that FGF2 signaling contributes to ICI-triggered HPD and the expression of IFN-γ/FGF-2/β-catenin was consistent [190]. In conclusion, mechanistically, IFN-γ secreted by T-cells promotes HPD by acting on FGF-2-PKM2-β-catenin signal transduction in tumor cells [190].

Paradoxically, murine models of gastric cancer point to an improved immunotherapeutic effect of FGFR1 expression [54]. Analysis of clinical data noted that melanoma patients with FGFR mutations treated with ICI had better survival than those without mutations (median OS: 60.00 months vs. 31.00 months; HR: 0.58, 95% CI: 0.42–0.80; *P* = 0.0051) [193]. Meanwhile, a retrospective analysis noted that patients with non-small cell lung cancer carrying FGFR4 alterations had better objective remission rates (ORR) (50.0% vs. 19.4%; *P* = 0.057) and longer median PFS to immunotherapy (13.17 vs. 3.17 months; HR 0.37; 95% CI 0.14–1; *P* = 0.04) [194]. However, in urothelial carcinoma, some evidence indicate that the remission and survival rates after ICB monotherapy are independent of whether *FGFR* is mutated or not [20, 66, 195–197]. Real-world retrospective studies evaluating the effect of ICB therapy in patients with mutated *FGFR3* and WT *FGFR3* tumors observed no significant difference in OS or PFS between the two [66]. Analysis of two phase 2 clinical trials, Checkmate 275 and IMVigor 210, revealed no statistically significant differences in ICB treatment response rates or OS in patients with or without *FGFR3* mutations [20]. This may be due to the fact that *FGFR* mutations reduce mesenchymal-mediated immunosuppression, which counteracts the immunosuppression caused by low levels of T-cell infiltration in *FGFR*-mutated tumors [20, 198].

In conclusion, multiple outcomes exist for FGFR dysregulation on immune monotherapy. We summarized the different immunotherapy outcomes caused by FGF/FGFR signaling in Table 2. At present, there is a lack of sufficient evidence to indicate that FGFR signaling can be used as a specific marker for the effectiveness of ICB therapy.

**Table 2:** Relationship between FGF/FGFR dysregulation and immunotherapy

	Tumor	Data sources	Mutation information	Results of immune monotherapy	Refs
FGFR dysregulation reduces immunotherapy efficacy	Locally advanced and unresectable or metastatic UC	NCT02365597	FGFR Mutations /Fusions	1 of 22 patients (5%) responded to previous immunotherapy	[9]
	Melanoma	GSE78220	FGFR1/FGFR3 +	High FGFR1 + pts have Shorter OS; FGFR3 + pts have poorer PFS	[111, 204]
	Advanced UC	Real world	FGFR+	OS: FGFR+/FGFR- (3.1 mo vs. 6.1 mo, HR 1.33, 95% CI 0.78–2.26, p = 0.30)	[184]
	Advanced UC	NCT03390504	FGFR+	FGFR + pts have shorter OS and lower ORR and DCR	[186]
	NSCLC, Esophageal carcinoma etc.	Real world	FGF3/4/19 amplification	Tumor hyperprogression	[189]
FGFR dysregulation enhances immunotherapy efficacy	Melanoma	Real world	FGFR Mutations	FGFR + pts have better mOS and higher ORR	[193]
	Nonsmall cell lung cancer	Real world	FGFR4-altered	FGFR4-altered pts have better ORR and longer mPFS	[194]
FGFR dysregulation not associated with immunotherapy efficacy	Metastatic Advanced UC	IMvigor 210 + CheckMate 275	FGFR3 Mutations	There was no statistically significant difference in response rate or OS	[20]
	Muscle-invasive Urothelial Bladder Carcinoma	NCT02736266.	FGFR3-altered	No correlation was found between FGFR3 activity or mutations/fusions and CR	[195]
	UC	Real Worldv	FGFR3-altered	ORR:FGFR3-altered/FGFR-wild(12% vs. 19%, p = 0.73)	[66]

UC, urothelial carcinoma; +, positive; -, negative; pts, patients; OS, overall survival; mOS, median overall survival; PFS, progression-free survival; mPFS, median progression-free survival; mo, month; HR, hazard ratio; CI, confidence interval; ORR, objective response rate; DCR, disease control rate; CR, complete response

### Effectiveness of FGFR inhibitors in combination with ICB therapy

Despite controversial findings surrounding ICB monotherapy in *FGFR*-mutated tumors, most preclinical trial results indicate that FGFR-TKI combined with ICB therapy can effectively enhance the antitumor effect in *FGFR*-mutated tumors compared with any monotherapy [22, 23, 55, 144]. Evidence suggests that inadequate T-cell activation and inappropriate TME will impede the normal tumor-killing function of T-cells and thus diminish ICB efficacy [170]. Successful antitumor immune responses accompanied by immune checkpoint blockade require reactivation and clonal proliferation of antigen-experienced T-cells in the TME [170, 173, 199]. FGFR-TKI may assist ICB treatment through tumor immune circulation compared with anti-PD-1 treatment alone and CD8^+^ T-cells secrete more IFN-γ and GZMB after combination treatment with lenvatinib, which induces a more intense anti-tumor response [24, 62]. Lenvatinib co-treatment with anti-PD-1 therapy also leads to long-term immune memory formation while synergistically regulating the TME and tumor vascular normalization and enhancing the cytotoxic effects of T-cells, promoting therapeutic efficacy in HCC [22]. In a mouse model of lung cancer, FGFR-TKI enhanced the antitumor effects of PD-1/PD-L1 inhibitors by increasing T-cell infiltration and inducing ICD [23]. Analysis of multiple mouse models of bladder cancer showed that combination treatment with erdafitinib and anti-PD-1 antibodies increased the proportion and activation of CD8^+^ T-cells [25]. Moreover, the combination treatment group achieved the best results in both prolonging survival and inhibiting tumor growth compared to the control group [25].

Given the remarkable results achieved in preclinical studies of the combination of FGFR-TKI and ICI, relevant clinical trials are in full swing. In Table 3, we summarize the clinical trials of ICB in combination with FGFR-TKI, the majority of which are still in phase 1/2 of clinical trials. The only phase 3 trial (ClinicalTrials.gov number, NCT05111626) will also undergo a phase 1b safety evaluation prior before the trial. This phase 3 trial was designed to compare the efficacy of chemotherapy plus immunotherapy with that of FGFR-TKI plus immune plus chemotherapy in advanced gastric and gastroesophageal junction cancer with FGFR2b overexpression. Regardless of the results, it will have specific implications for guiding clinical treatment. In addition, an early phase clinical trial (ClinicalTrials.gov number, NCT02325739), for which results are available, evaluated the safety of the combination therapy ICB [52]. The results indicated the safety of biologically active doses of FGF401 (an FGFR4 inhibitor) alone or in combination with spartalizumab (an anti-PD-1 antibody) in the treatment of FGFR4/KLB-positive tumors. In addition, other studies have noted that the combination was generally well tolerated with acceptable side effects [200–202]. Notably, in a pivotal trial of erdafitinib(ClinicalTrials.gov number, NCT02365597), response rates were higher in patients with prior ICB treatment compared to the entire cohort (59% vs. 40%) [9]. Other clinical trials (ClinicalTrials.gov numbers, NCT04604132, NCT04045613, and NCT04003610) have been completed and await publication of the final results. In the real world, the efficacy of combination therapy has also been supported. In breast cancer patients, complete remission in 60% of the combination treatment group in breast cancer patients and there was a significant increase in CD4^+^ and CD8^+^ T-cell infiltration and a decrease in MDSCs, M1-type macrophages, and Treg cell infiltration were observed [111]. However, some clinical study data suggest that FGFR-ICB combination therapy does not achieve a significant benefit [144, 203]. This contradictory finding of FGFR-ICB therapy in combination with ICB in terms of efficacy has been suggested to be related to different tumor staging and certain biomarkers [144].

**Table 3:** Clinical trials of FGFR-TKI combination immunotherapy

NCT Number	Phases	Status	Tumor	Intervention1	Intervention 2	Intervention 3	Intervention 4	Treatment line	Gene state
NCT05614739	Phase 1	Recruiting	Urinary Bladder Neoplasms Neoplasm Metastasis Ureteral Neoplasms	LOXO-435	LOXO-435 +Pembrolizumab			After standard therapies	FGFR3-altered
NCT04601857	Phase 2	Recruiting	Advanced and Metastatic Urothelial Cancer	Futibatinib +Pembrolizumab				First-line	FGFR3 Mutation; FGFR1-4 Fusion/Rearrangement
NCT02401542	Phase 1/2	Terminated	Locally Advanced or Metastatic Urothelial Cell Carcinoma Urinary Bladder Disease Urological Diseases	Vofatamab +Docetaxel	Docetaxel +Placebo	Vofatamab		Second or subsequent	FGFR3 Mutant/Fusion
NCT05267470	Phase 1	Recruiting	Squamous-Cell Non-Small-Cell Lung Cancer	Bemarituzumab +Docetaxel	Bemarituzumab	Bemarituzumab +Pembrolizumab +Carboplatin +Paclitaxel		Intervention 1: Second or subsequent Intervention2: Third or subsequent Intervention3: First or subsequent	FGFR2b Overexpression
NCT05564416	Phase 2	Recruiting	Localized Bladder Cancer	Erdafitinib	Erdafitinib +Atezolizumab			Neoadjuvant	FGFR2/3 Alterations
NCT04604132	Phase 1/2	Completed	Gastric Adenocarcinoma	Derazantinib	Derazantinib +Paclitaxel +Ramucirumab	Derazantinib +Atezolizumab	Paclitaxel +Ramucirumab	Second or subsequent	FGFR2 Fusions/Rearrangements/Amplifications; FGFR1-3 Mutations/Short variants
NCT05174650	Phase 2	Recruiting	Intrahepatic Cholangiocarcinoma	Atezolizumab +Derazantinib				First or Second line	FGFR2 Fusions/Rearrangements
NCT05510427	Phase 1	Not yet recruiting	Cholangiocarcinoma Liver Cancer	Infigratinib +Atezolizumab +Bevacizumab				Second or subsequent	FGFR2 Fusion/Amplification
NCT04045613	Phase 1/2	Completed	Urothelial Carcinoma	Derazantinib	Derazantinib +Atezolizumab	Derazantinib +/-Atezolizumab		Intervention 1: Second or subsequent Intervention2: First-line Intervention3: Second-line	FGFR1-3 Mutations/Short variants and Rearrangements/fusions
NCT05004974	Phase 2	Not yet recruiting	Advanced Non Small Cell Lung Cancer	Sintilimab +Pemigatinib				First-line	FGFR Mutation
NCT03473743	Phase 1/2	Active, not recruiting	Urothelial Carcinoma	Erdafitinib +Cetrelimab	Erdafitinib +Cetrelimab +Cisplatin/Carboplatin	Erdafitinib		Intervention 1: Any line Intervention2/3: First-line	FGFR Alterations
NCT04828486	Phase 2	Recruiting	Advanced Hepatocellular Carcinoma	Futibatinib +Pembrolizumab				Second-line	FGF-19 Positive
NCT03473756	Phase 1b/2	Active, not recruiting	Locally Advanced or Metastatic Urothelial Carcinoma	Rogaratinib + Atezolizumab				First-line	FGFR1/3 mRNA expression
NCT02325739	Phase 1/2	Completed	Solid Malignancies	FGF401	FGF401 +PDR001			Intervention 1: After standard therapies Intervention2: Third or subsequent	
NCT02925533	Phase 1	Terminated	Bladder Cancer	Vofatamab +Pembrolizumab				Second or subsequent	
NCT03123055	Phase 1/2	Terminated	Locally Advanced or Metastatic Urothelial Cell Carcinoma	Vofatamab	Vofatamab +Pembrolizumab			First-line	
NCT04003610	Phase 2	Terminated	Metastatic Urothelial Carcinoma Unresectable Urothelial Carcinoma	Pemigatinib +Pembrolizumab	Pembrolizumab	Gemcitabine +Carboplatin		First-line	
NCT04699643	Phase 1/2	Recruiting	Advanced Solid Tumors	EVER4010001 +Pembrolizumab				After standard therapies	
NCT05111626	Phase 3	Recruiting	Gastric Cancer Gastroesophageal Junction Adenocarcinoma	Bemarituzumab +Nivolumab +mFOLFOX6	Nivolumab +mFOLFOX6 +Placebo			First-line	
NCT05173142	Phase 1/2	Recruiting	Solid Tumor	HMPL-453 monotherapy	HMPL-453 + chemotherapy /anti-PD-1 mAb			After standard therapies	
NCT05253053	Phase 1/2	Recruiting	Advanced Solid Tumor	TT-00420	TT-00420 +Atezolizumab	TT-00420 +nab-paclitaxel		After standard therapies	
NCT05322577	Phase 1	Recruiting	Gastric Cancer Gastroesophageal Junction Cancer	Bemarituzumab +CAPOX	Bemarituzumab +CAPOX +Nivolumab	Bemarituzumab +SOX +Nivolumab		First-line	
NCT05369286	Phase 1	Recruiting	Solid Tumor	MAX-40279-01 +Toripalimab					

In conclusion, although certain preclinical studies suggest that the combination of FGFR-TKI and ICB treatment may promote antitumor effects, this finding has only been demonstrated in a small number of malignancies treated in clinical trials, and direct results have not been observed in other tumors. Related clinical trials are also advancing in a variety of malignancies, and more clinical results are expected to conclude the efficacy of the combination therapy.

## Conclusion and perspective

Aberrant regulation of FGFR signaling assists in tumor generation and further promotes tumor cell proliferation and survival. In this review, we summarize the specific roles of the FGFR signaling pathway in the TME, noting that FGFR signaling shapes immunosuppressive TME by promoting non-T-cell immune infiltration, recruitment and survival of MDSCs and TAMs, EMT, and angiogenesis, leading to immune escape and tumor metastasis. In contrast, FGFR-TKI therapy can reverse the immunosuppressive microenvironment by promoting IFN-γ and GZMB secretion, inhibiting PD-1/PD-L1 and MHC expression, suppressing chemokine production, and promoting normalization of the vascular system. We further discussed the relationship between *FGFR* alteration and ICB treatment, drawing contradictory conclusions. In addition, we highlight that FGFR-TKI may aid ICB therapy with the help of cancer immune circulation (e.g., assisting immune cell transport and infiltration, promoting T-cell initiation, reducing immunosuppressive cells, and elevating tumor antigenicity). Although the findings of certain clinical trials do not seem to support the effectiveness of FGFR-TKI in combination therapy with ICB, further analysis of additional studies and data is required.

Of course, there are still some questions worth thinking about and exploring. First, the emergence of drug resistance during the treatment of oncology patients is an unavoidable topic. Analyzing the mechanism of FGFR-TKI resistance will bring benefits to clinical treatment. Previous studies have also pointed out that the co-expression of different FGFRs, the expression of Klothoβ, and the activation of bypass signaling are associated with FGFR-TKI resistance. In TME, stromal or tumor cells can induce the development of drug resistance by secreting soluble factors [204]. However, the state of the microenvironment in FGFR-TKI-resistant patients has not been clearly demonstrated, and the exploration of the correlation between the development of FGFR-TKI resistance and the microenvironment is also promising. For FGFR-TKI-resistant patients, whether the therapeutic effect after combination immunotherapy can be restored or improved is also a topic worth discussing. Currently, the mechanism of FGFR-TKI and immunotherapy combination in tumors has not been clearly explored and more relevant explorations should be performed, such as single-cell sequencing, Organoid-immune cell co-culture model, more relevant clinical trials, etc. Secondly, in our article and existing clinical trials, the combination of FGFR-TKI with ICI therapy is basically anti-PD-(L)1 therapy. There is no evidence to support the combination therapy of other immune checkpoints (e.g., anti-CTLA4, LAG3, etc.).

In the past decade, immunotherapy has made significant breakthroughs in improving the prognosis of cancer patients, and we believe that FGFR inhibitors have great potential in the treatment of tumors in the future. To expand and prolong the benefits of FGFR inhibitors for patients, we need a better understanding of primary and secondary resistance. The development of combination protocols and next-generation inhibitors that can delay or overcome drug resistance may improve the efficacy of current FGFR-signaling pathway-based tumor treatment regimens.

## Data Availability

Not applicable.

## Abbreviations

APCs: Antigen-presenting cells
BMDMs: Bone marrow-derived macrophage
CAFs: Cancer associated fibroblasts
CAMs: Cell adhesion molecules
CIITA: MHC Class II transactivator
CTL: Cytotoxic T-lymphocytes
CTLA-4: Cytotoxic T lymphocyte-associated protein 4
DAMPs: Damage-associated molecular patterns
DC: Dendritic cells
ECM: Extracellular matrix
EMT: Epithelial-mesenchymal transition
FGF: Fibroblast growth factor
FGFBP: FGF binding protein
FGFR: Fibroblast growth factor receptor
FGFR-TKI: FGFR-tyrosine kinase inhibitors
FLRT3: Fibrillin leucine-rich transmembrane protein 3
FRS2: FGFR substrate 2
G-CSF: Decreases granulocyte colony-stimulating factor
GEMM: Genetically engineered mouse models
GM-CSF: Granulocyte-macrophage colony-stimulating factor
GPCRs: G protein-coupled receptors
GZMB: Granzyme B
HCC: Hepatocellular carcinoma
HK2: Hexokinase 2
HLA: Human leukocyte antigen
HNSCC: Head and neck squamous cell carcinoma
HPSG: Heparan-sulfate proteoglycans
HS: Heparan sulfate
ICAM1: Intercellular cell adhesion molecule 1
ICC: Intrahepatic cholangiocarcinoma
ICD: Immunogenic tumor death
ICB: Immune checkpoint blockade
IFN-γ: Interferon-γ
IFP: Interstitial fluid pressure
L1CAM: L1 cell adhesion molecule
LECs: Lymphatic endothelial cells
MHC: Major histocompatibility complex
MKP3: MAPK phosphatase 3
MMP2: Matrix metalloproteinase 2
mTOR: Mammalian target of rapamycin
MDSCs: Myeloid-derived suppressor cells
NK: Natural killer
OS: Overall survival
PD-1: Programmed cell death protein 1
PFS: Progression-free survival
PLCγ: Phospholipase C
RSK2: Ribosomal protein S6 kinase 2
RTK: Receptor tyrosine kinase
SEF: Similar expression to FGF
SOSC1: Suppressor of cytokine signaling 1
STAT: Signal transducer and activator of transcription
TAM: Tumor-associated macrophage
TGF-β1: Transforming growth factor β1
Th: T helper
TILs: Tumor-infiltrating lymphocytes
TLR2: Toll-like receptor 2
TME: Tumor microenvironment
Treg: Regulatory T
VCAM1: Vascular cell adhesion molecule 1
WT: Wildtype

## References

[1] Ornitz DM, Itoh N (2015). The fibroblast growth factor signaling pathway. Wires Dev Biol.

[2] Xie Y. FGF/FGFR signaling in health and disease.Signal Transduction and Targeted Therapy. 2020;38. 10.1038/s41392-020-00222-7 PMC7468161 32879300

[3] Turner N, Grose R (2010). Fibroblast growth factor signalling: from development to cancer. Nat Rev Cancer.

[4] Katoh M, Nakagama H (2014). FGF receptors: cancer biology and therapeutics. Med Res Rev.

[5] Carter EP, Fearon AE, Grose RP (2015). Careless talk costs lives: fibroblast growth factor receptor signalling and the consequences of pathway malfunction. Trends Cell Biol.

[6] Babina IS, Turner NC (2017). Advances and challenges in targeting FGFR signalling in cancer. Nat Rev Cancer.

[7] Katoh M (2019). Fibroblast growth factor receptors as treatment targets in clinical oncology. Nat Rev Clin Oncol.

[8] Tao Z, Cui Y, Xu X, Han T (2022). FGFR redundancy limits the efficacy of FGFR4-selective inhibitors in hepatocellular carcinoma. Proc Natl Acad Sci USA.

[9] Loriot Y, Necchi A, Park SH, Garcia-Donas J, Huddart R, Burgess E (2019). Erdafitinib in locally Advanced or Metastatic Urothelial Carcinoma. N Engl J Med.

[10] Fourcade J, Sun Z, Benallaoua M, Guillaume P, Luescher IF, Sander C (2010). Upregulation of Tim-3 and PD-1 expression is associated with tumor antigen-specific CD8 + T cell dysfunction in melanoma patients. J Exp Med.

[11] Woo S-R, Turnis ME, Goldberg MV, Bankoti J, Selby M, Nirschl CJ (2012). Immune inhibitory molecules LAG-3 and PD-1 synergistically regulate T-cell function to promote tumoral immune escape. Cancer Res.

[12] Fs H, Sj O, Df M, Rw W, Ja S, Jb H et al. Improved survival with ipilimumab in patients with metastatic melanoma. The New England journal of medicine [Internet]. N Engl J Med; 2010 [cited 2023 Feb 6];363. Available from: https://pubmed.ncbi.nlm.nih.gov/20525992/10.1056/NEJMoa1003466 PMC3549297 20525992

[13] Chae YK, Oh MS, Giles FJ (2018). Molecular biomarkers of primary and Acquired Resistance to T-Cell-mediated immunotherapy in Cancer: Landscape, Clinical Implications, and future directions. Oncologist.

[14] Gainor JF, Shaw AT, Sequist LV, Fu X, Azzoli CG, Piotrowska Z (2016). EGFR mutations and ALK rearrangements are Associated with low response rates to PD-1 pathway blockade in Non-Small Cell Lung Cancer: a retrospective analysis. Clin Cancer Res.

[15] Katoh M (2016). FGFR inhibitors: Effects on cancer cells, tumor microenvironment and whole-body homeostasis (review). Int J Mol Med.

[16] Chen DS, Mellman I (2013). Oncology meets immunology: the Cancer-Immunity cycle. Immunity.

[17] Shono T, Kanetake H, Kanda S (2001). The role of mitogen-activated protein kinase activation within focal adhesions in chemotaxis toward FGF-2 by murine brain capillary endothelial cells. Exp Cell Res.

[18] Yu P, Wilhelm K, Dubrac A, Tung JK, Alves TC, Fang JS (2017). FGF-dependent metabolic control of vascular development. Nature.

[19] Reed JR, Stone MD, Beadnell TC, Ryu Y, Griffin TJ, Schwertfeger KL. Fibroblast Growth Factor Receptor 1 Activation in Mammary Tumor Cells Promotes Macrophage Recruitment in a CX3CL1-Dependent Manner. Li Y, editor. PLoS ONE. 2012;7:e45877. 10.1371/journal.pone.0045877 PMC3454319 23029290

[20] Wang L, Gong Y, Saci A, Szabo PM, Martini A, Necchi A (2019). Fibroblast growth factor receptor 3 alterations and response to PD-1/PD-L1 blockade in patients with metastatic Urothelial Cancer. Eur Urol.

[21] Sweis RF, Spranger S, Bao R, Paner GP, Stadler WM, Steinberg G (2016). Molecular drivers of the Non-T-cell-Inflamed Tumor Microenvironment in urothelial bladder Cancer. Cancer Immunol Res.

[22] Deng H, Kan A, Lyu N, Mu L, Han Y, Liu L (2020). Dual vascular endothelial growth factor receptor and fibroblast growth factor receptor inhibition elicits Antitumor Immunity and enhances programmed cell Death-1 checkpoint blockade in Hepatocellular Carcinoma. Liver Cancer.

[23] Palakurthi S, Kuraguchi M, Zacharek SJ, Zudaire E, Huang W, Bonal DM (2019). The combined effect of FGFR inhibition and PD-1 blockade promotes tumor-intrinsic induction of Antitumor Immunity. Cancer Immunol Res.

[24] Yi C, Chen L, Lin Z, Liu L, Shao W, Zhang R (2021). Lenvatinib targets FGF receptor 4 to enhance Antitumor Immune response of Anti-Programmed Cell Death-1 in HCC. Hepatology.

[25] Jing W, Wang G, Cui Z, Xiong G, Jiang X, Li Y (2022). FGFR3 destabilizes PD-L1 via NEDD4 to control T-cell-mediated bladder Cancer Immune Surveillance. Cancer Res.

[26] Itoh N, Ornitz DM (2011). Fibroblast growth factors: from molecular evolution to roles in development, metabolism and disease. J Biochem.

[27] Korsensky L, Ron D (2016). Regulation of FGF signaling: recent insights from studying positive and negative modulators. Semin Cell Dev Biol.

[28] Eswarakumar VP, Lax I, Schlessinger J (2005). Cellular signaling by fibroblast growth factor receptors. Cytokine Growth Factor Rev.

[29] Sleeman M, Fraser J, McDonald M, Yuan S, White D, Grandison P (2001). Identification of a new fibroblast growth factor receptor, FGFR5. Gene.

[30] Wiedemann M, Trueb B (2000). Characterization of a novel protein (FGFRL1) from human cartilage related to FGF receptors. Genomics.

[31] Kang S, Elf S, Dong S, Hitosugi T, Lythgoe K, Guo A (2009). Fibroblast growth factor receptor 3 associates with and tyrosine phosphorylates p90 RSK2, leading to RSK2 activation that mediates hematopoietic transformation. Mol Cell Biol.

[32] Lax I, Wong A, Lamothe B, Lee A, Frost A, Hawes J (2002). The docking protein FRS2alpha controls a MAP kinase-mediated negative feedback mechanism for signaling by FGF receptors. Mol Cell.

[33] Wong A, Lamothe B, Lee A, Schlessinger J, Lax I, Li A (2002). FRS2 alpha attenuates FGF receptor signaling by Grb2-mediated recruitment of the ubiquitin ligase Cbl. Proc Natl Acad Sci U S A.

[34] N MLAC et al. P, M K, J O, M Z,. Cross-Talk between Fibroblast Growth Factor Receptors and Other Cell Surface Proteins. Cells [Internet]. Cells; 2019 [cited 2022 Jun 26];8. Available from: https://pubmed.ncbi.nlm.nih.gov/31091809/10.3390/cells8050455 PMC6562592 31091809

[35] Qian X, Anzovino A, Kim S, Suyama K, Yao J, Hulit J (2014). N-cadherin/FGFR promotes metastasis through epithelial-to-mesenchymal transition and stem/progenitor cell-like properties. Oncogene.

[36] Cattaneo F, Guerra G, Parisi M, De Marinis M, Tafuri D, Cinelli M (2014). Cell-surface receptors transactivation mediated by g protein-coupled receptors. Int J Mol Sci.

[37] Di Liberto V, Mudò G, Belluardo N (2019). Crosstalk between receptor tyrosine kinases (RTKs) and G protein-coupled receptors (GPCR) in the brain: focus on heteroreceptor complexes and related functional neurotrophic effects. Neuropharmacology.

[38] Yokote H, Fujita K, Jing X, Sawada T, Liang S, Yao L (2005). Trans-activation of EphA4 and FGF receptors mediated by direct interactions between their cytoplasmic domains. Proc Natl Acad Sci U S A.

[39] Chen P-Y, Simons M, Friesel R (2009). FRS2 via fibroblast growth factor receptor 1 is required for platelet-derived growth factor receptor beta-mediated regulation of vascular smooth muscle marker gene expression. J Biol Chem.

[40] Tassi E, Al-Attar A, Aigner A, Swift MR, McDonnell K, Karavanov A (2001). Enhancement of fibroblast growth factor (FGF) activity by an FGF-binding protein. J Biol Chem.

[41] Schulze D, Plohmann P, Höbel S, Aigner A (2011). Anti-tumor effects of fibroblast growth factor-binding protein (FGF-BP) knockdown in colon carcinoma. Mol Cancer.

[42] Böttcher RT, Pollet N, Delius H, Niehrs C (2004). The transmembrane protein XFLRT3 forms a complex with FGF receptors and promotes FGF signalling. Nat Cell Biol.

[43] Beenken A, Mohammadi M (2009). The FGF family: biology, pathophysiology and therapy. Nat Rev Drug Discov.

[44] Helsten T, Elkin S, Arthur E, Tomson BN, Carter J, Kurzrock R (2016). The FGFR Landscape in Cancer: analysis of 4,853 tumors by Next-Generation sequencing. Clin Cancer Res.

[45] Im JH, Buzzelli JN, Jones K, Franchini F, Gordon-Weeks A, Markelc B (2020). FGF2 alters macrophage polarization, tumour immunity and growth and can be targeted during radiotherapy. Nat Commun.

[46] Kim RD, Sarker D, Meyer T, Yau T, Macarulla T, Park J-W (2019). First-in-human phase I study of Fisogatinib (BLU-554) validates aberrant FGF19 signaling as a driver event in Hepatocellular Carcinoma. Cancer Discov.

[47] Sridharan V, Neyaz A, Chougule A, Baiev I, Reyes S, Barr Fritcher EG et al. FGFR mRNA Expression in Cholangiocarcinoma and its Correlation with *FGFR2* Fusion Status and Immune Signatures.Clinical Cancer Research. 2022;CCR-22-1244. 10.1158/1078-0432.CCR-22-1244 PMC9751751 36190545

[48] Katoh M, Therapeutics Targeting FGF (2016). Signaling Network in Human Diseases. Trends Pharmacol Sci.

[49] Holdman XB, Welte T, Rajapakshe K, Pond A, Coarfa C, Mo Q (2015). Upregulation of EGFR signaling is correlated with tumor stroma remodeling and tumor recurrence in FGFR1-driven breast cancer. Breast Cancer Res.

[50] Rossaint J, Oehmichen J, Aken HV, Reuter S, Pavenstädt HJ, Meersch M (2016). FGF23 signaling impairs neutrophil recruitment and host defense during CKD. J Clin Invest American Society for Clinical Investigation.

[51] Han X, Li L, Yang J, King G, Xiao Z, Quarles LD (2016). Counter-regulatory paracrine actions of FGF-23 and 1,25(OH)2D in macrophages. FEBS Lett.

[52] Chan SL, Schuler M, Kang Y-K, Yen C-J, Edeline J, Choo SP (2022). A first-in-human phase 1/2 study of FGF401 and combination of FGF401 with spartalizumab in patients with hepatocellular carcinoma or biomarker-selected solid tumors. J Exp Clin Cancer Res.

[53] Bejarano L, Jordāo MJC, Joyce JA (2021). Therapeutic targeting of the Tumor Microenvironment. Cancer Discov.

[54] Yang C, Song D, Zhao F, Wu J, Zhang B, Ren H (2022). Comprehensive analysis of the prognostic value and immune infiltration of FGFR family members in gastric cancer. Front Oncol.

[55] Robinson BD, Vlachostergios PJ, Bhinder B, Liu W, Li K, Moss TJ (2019). Upper tract urothelial carcinoma has a luminal-papillary T-cell depleted contexture and activated FGFR3 signaling. Nat Commun.

[56] Franco F, Jaccard A, Romero P, Yu Y-R, Ho P-C (2020). Metabolic and epigenetic regulation of T-cell exhaustion. Nat Metab.

[57] Thommen DS, Schumacher TN (2018). T cell dysfunction in Cancer. Cancer Cell.

[58] Gocher AM, Workman CJ, Vignali DAA (2022). Interferon-γ: teammate or opponent in the tumour microenvironment?. Nat Rev Immunol.

[59] Kono M, Komatsuda H, Yamaki H, Kumai T, Hayashi R, Wakisaka R (2022). Immunomodulation via FGFR inhibition augments FGFR1 targeting T-cell based antitumor immunotherapy for head and neck squamous cell carcinoma. OncoImmunology.

[60] Necchi A, Joseph RW, Loriot Y, Hoffman-Censits J, Perez-Gracia JL, Petrylak DP (2017). Atezolizumab in platinum-treated locally advanced or metastatic urothelial carcinoma: post-progression outcomes from the phase II IMvigor210 study. Ann Oncol.

[61] Sharma P, Retz M, Siefker-Radtke A, Baron A, Necchi A, Bedke J (2017). Nivolumab in metastatic urothelial carcinoma after platinum therapy (CheckMate 275): a multicentre, single-arm, phase 2 trial. Lancet Oncol.

[62] Kato Y, Tabata K, Kimura T, Yachie-Kinoshita A, Ozawa Y, Yamada K (2019). Lenvatinib plus anti-PD-1 antibody combination treatment activates CD8 + T cells through reduction of tumor-associated macrophage and activation of the interferon pathway. PLoS ONE.

[63] Adachi Y, Kamiyama H, Ichikawa K, Fukushima S, Ozawa Y, Yamaguchi S (2022). Inhibition of FGFR reactivates IFNγ Signaling in Tumor cells to enhance the combined antitumor activity of Lenvatinib with Anti-PD-1 antibodies. Cancer Res.

[64] Korpal M, Puyang X, Jeremy Wu Z, Seiler R, Furman C, Oo HZ (2017). Evasion of immunosurveillance by genomic alterations of PPARγ/RXRα in bladder cancer. Nat Commun.

[65] Rochel N, Krucker C, Coutos-Thévenot L, Osz J, Zhang R, Guyon E (2019). Recurrent activating mutations of PPARγ associated with luminal bladder tumors. Nat Commun.

[66] Rose TL, Weir WH, Mayhew GM, Shibata Y, Eulitt P, Uronis JM (2021). Fibroblast growth factor receptor 3 alterations and response to immune checkpoint inhibition in metastatic urothelial cancer: a real world experience. Br J Cancer.

[67] Kardos J, Chai S, Mose LE, Selitsky SR, Krishnan B, Saito R (2016). Claudin-low bladder tumors are immune infiltrated and actively immune suppressed. JCI Insight.

[68] Byrd V, Zhao XM, McKeehan WL, Miller GG, Thomas JW (1996). Expression and functional expansion of fibroblast growth factor receptor T cells in rheumatoid synovium and peripheral blood of patients with rheumatoid arthritis. Arthritis Rheum.

[69] Byrd VM, Ballard DW, Miller GG, Thomas JW (1999). Fibroblast growth factor-1 (FGF-1) enhances IL-2 production and nuclear translocation of NF-kappaB in FGF receptor-bearing Jurkat T cells. J Immunol.

[70] Zhao XM, Byrd VM, McKeehan WL, Reich MB, Miller GG, Thomas JW (1995). Costimulation of human CD4 + T cells by fibroblast growth factor-1 (acidic fibroblast growth factor). J Immunol.

[71] Wang JK, Xu H, Li HC, Goldfarb M (1996). Broadly expressed SNT-like proteins link FGF receptor stimulation to activators of ras. Oncogene.

[72] Kouhara H, Hadari YR, Spivak-Kroizman T, Schilling J, Bar-Sagi D, Lax I (1997). A lipid-anchored Grb2-binding protein that links FGF-receptor activation to the Ras/MAPK signaling pathway. Cell.

[73] Rock KL, Reits E, Neefjes J (2016). Present yourself! By MHC class I and MHC class II molecules. Trends Immunol.

[74] Wieczorek M, Abualrous ET, Sticht J, Álvaro-Benito M, Stolzenberg S, Noé F (2017). Major histocompatibility complex (MHC) class I and MHC class II proteins: conformational plasticity in Antigen Presentation. Front Immunol.

[75] Masternak K, Muhlethaler-Mottet A, Villard J, Zufferey M, Steimle V, Reith W (2000). CIITA is a transcriptional coactivator that is recruited to MHC class II promoters by multiple synergistic interactions with an enhanceosome complex. Genes Dev.

[76] Steimle V, Siegrist CA, Mottet A, Lisowska-Grospierre B, Mach B (1994). Regulation of MHC class II expression by interferon-gamma mediated by the transactivator gene CIITA. Science.

[77] Pennini ME, Pai RK, Schultz DC, Boom WH, Harding CV (2006). Mycobacterium tuberculosis 19-kDa lipoprotein inhibits IFN-gamma-induced chromatin remodeling of MHC2TA by TLR2 and MAPK signaling. J Immunol.

[78] Pollack BP, Sapkota B, Cartee TV (2011). Epidermal growth factor receptor inhibition augments the expression of MHC class I and II genes. Clin Cancer Res.

[79] H W, B L, J W. Beta2-microglobulin(B2M) in cancer immunotherapies: Biological function, resistance and remedy. Cancer letters [Internet]. Cancer Lett; 2021 [cited 2022 Sep 15];517. Available from: https://pubmed.ncbi.nlm.nih.gov/34129878/10.1016/j.canlet.2021.06.008 34129878

[80] Pitt JM, Marabelle A, Eggermont A, Soria J-C, Kroemer G, Zitvogel L (2016). Targeting the tumor microenvironment: removing obstruction to anticancer immune responses and immunotherapy. Ann Oncol.

[81] Fridman WH, Pagès F, Sautès-Fridman C, Galon J (2012). The immune contexture in human tumours: impact on clinical outcome. Nat Rev Cancer.

[82] Courtney AH, Lo W-L, Weiss A (2018). TCR Signaling: mechanisms of initiation and propagation. Trends Biochem Sci.

[83] Chen L, Flies DB (2013). Molecular mechanisms of T cell co-stimulation and co-inhibition. Nat Rev Immunol.

[84] Kim H-J, Cantor H (2014). The path to reactivation of antitumor immunity and checkpoint immunotherapy. Cancer Immunol Res.

[85] Zou W, Wolchok JD, Chen L (2016). PD-L1 (B7-H1) and PD-1 pathway blockade for cancer therapy: mechanisms, response biomarkers, and combinations. Sci Transl Med.

[86] McNiel EA, Tsichlis PN (2017). Analyses of publicly available genomics resources define FGF-2-expressing bladder carcinomas as EMT-prone, proliferative tumors with low mutation rates and high expression of CTLA-4, PD-1 and PD-L1. Signal Transduct Target Ther.

[87] Bogatyrova O, Mattsson JSM, Ross EM, Sanderson MP, Backman M, Botling J (2021). FGFR1 overexpression in non-small cell lung cancer is mediated by genetic and epigenetic mechanisms and is a determinant of FGFR1 inhibitor response. Eur J Cancer.

[88] Lu M, Wang K, Ji W, Yu Y, Li Z, Xia W (2022). FGFR1 promotes tumor immune evasion via YAP-mediated PD-L1 expression upregulation in lung squamous cell carcinoma. Cell Immunol.

[89] Li P, Huang T, Zou Q, Liu D, Wang Y, Tan X (2019). FGFR2 promotes expression of PD-L1 in Colorectal Cancer via the JAK/STAT3 signaling pathway. JI.

[90] Jusakul A, Cutcutache I, Yong CH, Lim JQ, Huang MN, Padmanabhan N (2017). Whole-genome and epigenomic landscapes of etiologically distinct subtypes of Cholangiocarcinoma. Cancer Discov.

[91] Maraz A, Takacs P, Lawson J, Santiago-Walker A, Pajor L, Sukosd F (2019). Correlation between FGFR mutation and PD-L1 expression of urinary bladder cancers: a real-world based biomarker study. JCO Wolters Kluwer.

[92] Roghmann F, Wirtz R, Jarczyk J, Kriegmair MC, Worst TS, Sikic D (2019). 933P - prognostic role of FGFR mutations and FGFR mRNA expression in metastatic urothelial cancer treated with anti-PD(L1) inhibitors in first and second-line setting. Ann Oncol.

[93] Rosenberg JE, Hoffman-Censits J, Powles T, van der Heijden MS, Balar AV, Necchi A (2016). Atezolizumab in patients with locally advanced and metastatic urothelial carcinoma who have progressed following treatment with platinum-based chemotherapy: a single-arm, multicentre, phase 2 trial. Lancet.

[94] Rosenberg JE, Gajate P, Morales-Barrera R, Lee J-L, Necchi A, Penel N (2020). Safety and preliminary efficacy of rogaratinib in combination with atezolizumab in a phase Ib/II study (FORT-2) of first-line treatment in cisplatin-ineligible patients (pts) with locally advanced or metastatic urothelial cancer (UC) and FGFR mRNA overexpression. JCO Wolters Kluwer.

[95] Cassetta L, Pollard JW (2018). Targeting macrophages: therapeutic approaches in cancer. Nat Rev Drug Discov.

[96] Hanahan D, Weinberg RA (2011). Hallmarks of cancer: the next generation. Cell.

[97] Condeelis J, Pollard JW (2006). Macrophages: obligate partners for tumor cell migration, invasion, and metastasis. Cell.

[98] Quail DF, Joyce JA (2013). Microenvironmental regulation of tumor progression and metastasis. Nat Med.

[99] Noy R, Pollard JW (2014). Tumor-associated macrophages: from mechanisms to therapy. Immunity.

[100] Schreiber RD, Old LJ, Smyth MJ (2011). Cancer immunoediting: integrating immunity’s roles in cancer suppression and promotion. Science.

[101] Talmadge JE, Gabrilovich DI (2013). History of myeloid-derived suppressor cells. Nat Rev Cancer.

[102] Mantovani A, Sica A (2010). Macrophages, innate immunity and cancer: balance, tolerance, and diversity. Curr Opin Immunol.

[103] Sakaguchi S, Miyara M, Costantino CM, Hafler DA (2010). FOXP3 + regulatory T cells in the human immune system. Nat Rev Immunol.

[104] Groth C, Hu X, Weber R, Fleming V, Altevogt P, Utikal J (2019). Immunosuppression mediated by myeloid-derived suppressor cells (MDSCs) during tumour progression. Br J Cancer.

[105] Marvel D, Gabrilovich DI (2015). Myeloid-derived suppressor cells in the tumor microenvironment: expect the unexpected. J Clin Invest.

[106] Bauer CA, Kim EY, Marangoni F, Carrizosa E, Claudio NM, Mempel TR (2014). Dynamic Treg interactions with intratumoral APCs promote local CTL dysfunction. J Clin Invest.

[107] de Visser KE, Eichten A, Coussens LM (2006). Paradoxical roles of the immune system during cancer development. Nat Rev Cancer.

[108] DeNardo DG, Barreto JB, Andreu P, Vasquez L, Tawfik D, Kolhatkar N (2009). CD4(+) T cells regulate pulmonary metastasis of mammary carcinomas by enhancing protumor properties of macrophages. Cancer Cell.

[109] Schwertfeger KL, Xian W, Kaplan AM, Burnett SH, Cohen DA, Rosen JM (2006). A critical role for the inflammatory response in a mouse model of preneoplastic progression. Cancer Res.

[110] Takase N, Koma Y-I, Urakawa N, Nishio M, Arai N, Akiyama H (2016). NCAM- and FGF-2-mediated FGFR1 signaling in the tumor microenvironment of esophageal cancer regulates the survival and migration of tumor-associated macrophages and cancer cells. Cancer Lett.

[111] Wu Y, Yi Z, Li J, Wei Y, Feng R, Liu J (2022). FGFR blockade boosts T cell infiltration into triple-negative breast cancer by regulating cancer-associated fibroblasts. Theranostics.

[112] Park MH, Lee JS, Yoon JH (2012). High expression of CX3CL1 by tumor cells correlates with a good prognosis and increased tumor-infiltrating CD8 + T cells, natural killer cells, and dendritic cells in breast carcinoma. J Surg Oncol.

[113] Liu L, Ye TH, Han YP, Song H, Zhang YK, Xia Y (2014). Reductions in myeloid-derived suppressor cells and lung metastases using AZD4547 treatment of a metastatic murine breast tumor model. Cell Physiol Biochem.

[114] Ye T, Wei X, Yin T, Xia Y, Li D, Shao B (2014). Inhibition of FGFR signaling by PD173074 improves antitumor immunity and impairs breast cancer metastasis. Breast Cancer Res Treat.

[115] Akhand SS, Liu Z, Purdy SC, Abdullah A, Lin H, Cresswell GM (2020). Pharmacologic inhibition of FGFR modulates the metastatic Immune Microenvironment and promotes response to Immune Checkpoint Blockade. Cancer Immunol Res.

[116] Welte T, Kim IS, Tian L, Gao X, Wang H, Li J (2016). Oncogenic mTOR signalling recruits myeloid-derived suppressor cells to promote tumour initiation. Nat Cell Biol.

[117] Matsumura S, Wang B, Kawashima N, Braunstein S, Badura M, Cameron TO (2008). Radiation-Induced CXCL16 release by breast Cancer cells attracts Effector T cells. J Immunol.

[118] Korbecki J, Bajdak-Rusinek K, Kupnicka P, Kapczuk P, Simińska D, Chlubek D (2021). The role of CXCL16 in the pathogenesis of Cancer and Other Diseases. Int J Mol Sci.

[119] Karakasheva TA, Waldron TJ, Eruslanov E, Kim S-B, Lee J-S, O’Brien S (2015). CD38-Expressing myeloid-derived suppressor cells promote Tumor Growth in a murine model of Esophageal Cancer. Cancer Res.

[120] Zhao W, Xu Y, Xu J, Wu D, Zhao B, Yin Z (2015). Subsets of myeloid-derived suppressor cells in hepatocellular carcinoma express chemokines and chemokine receptors differentially. Int Immunopharmacol.

[121] Di Pilato M, Kfuri-Rubens R, Pruessmann JN, Ozga AJ, Messemaker M, Cadilha BL (2021). CXCR6 positions cytotoxic T cells to receive critical survival signals in the tumor microenvironment. Cell.

[122] Matloubian M, David A, Engel S, Ryan JE, Cyster JG (2000). A transmembrane CXC chemokine is a ligand for HIV-coreceptor Bonzo. Nat Immunol.

[123] Pastushenko I, Blanpain C (2019). EMT Transition States during Tumor Progression and Metastasis. Trends Cell Biol.

[124] Brabletz T (2012). To differentiate or not–routes towards metastasis. Nat Rev Cancer.

[125] Aiello NM, Kang Y (2019). Context-dependent EMT programs in cancer metastasis. J Exp Med.

[126] Dongre A, Rashidian M, Reinhardt F, Bagnato A, Keckesova Z, Ploegh HL (2017). Epithelial-to-mesenchymal transition contributes to immunosuppression in breast carcinomas. Cancer Res.

[127] Chen L, Gibbons DL, Goswami S, Cortez MA, Ahn Y-H, Byers LA (2014). Metastasis is regulated via microRNA-200/ZEB1 axis control of tumour cell PD-L1 expression and intratumoral immunosuppression. Nat Commun.

[128] Lou Y, Diao L, Cuentas ERP, Denning WL, Chen L, Fan YH (2016). Epithelial-mesenchymal transition is Associated with a distinct Tumor Microenvironment including elevation of inflammatory signals and multiple Immune Checkpoints in Lung Adenocarcinoma. Clin Cancer Res.

[129] Bischoff J (2019). Endothelial-to-mesenchymal transition. Circ Res.

[130] Wendt MK, Taylor MA, Schiemann BJ, Sossey-Alaoui K, Schiemann WP (2014). Fibroblast growth factor receptor splice variants are stable markers of oncogenic transforming growth factor β1 signaling in metastatic breast cancers. Breast Cancer Res.

[131] Shirakihara T, Horiguchi K, Miyazawa K, Ehata S, Shibata T, Morita I (2011). TGF-β regulates isoform switching of FGF receptors and epithelial-mesenchymal transition. EMBO J.

[132] Brown WS, Tan L, Smith A, Gray NS, Wendt MK (2016). Covalent targeting of fibroblast growth factor receptor inhibits metastatic breast Cancer. Mol Cancer Ther.

[133] B R DR, Mr MNAMFB. T. Expression of the FGFR2 mesenchymal splicing variant in epithelial cells drives epithelial-mesenchymal transition. Oncotarget [Internet]. Oncotarget; 2016 [cited 2023 Jan 25];7. Available from: https://pubmed.ncbi.nlm.nih.gov/26713601/10.18632/oncotarget.6706 PMC4868697 26713601

[134] Ichise T, Yoshida N, Ichise H (2014). FGF2-induced Ras-MAPK signalling maintains lymphatic endothelial cell identity by upregulating endothelial-cell-specific gene expression and suppressing TGFβ signalling through Smad2. J Cell Sci.

[135] Chen P-Y, Qin L, Barnes C, Charisse K, Yi T, Zhang X (2012). FGF regulates TGF-β signaling and endothelial-to-mesenchymal transition via control of let-7 miRNA expression. Cell Rep.

[136] Sahai E, Astsaturov I, Cukierman E, DeNardo DG, Egeblad M, Evans RM (2020). A framework for advancing our understanding of cancer-associated fibroblasts. Nat Rev Cancer.

[137] Mao X, Xu J, Wang W, Liang C, Hua J, Liu J (2021). Crosstalk between cancer-associated fibroblasts and immune cells in the tumor microenvironment: new findings and future perspectives. Mol Cancer.

[138] X C. E S. Turning foes to friends: targeting cancer-associated fibroblasts. Nature reviews Drug discovery [Internet]. Nat Rev Drug Discov; 2019 [cited 2022 May 21];18. Available from: https://pubmed.ncbi.nlm.nih.gov/30470818/10.1038/s41573-018-0004-1 30470818

[139] Li C, Teixeira AF, Zhu H-J, Ten Dijke P (2021). Cancer associated-fibroblast-derived exosomes in cancer progression. Mol Cancer.

[140] Bohrer LR, Chuntova P, Bade LK, Beadnell TC, Leon RP, Brady NJ (2014). Activation of the FGFR–STAT3 pathway in breast Cancer cells induces a Hyaluronan-Rich Microenvironment that Licenses Tumor formation. Cancer Res.

[141] Schito L, Rey S, Hypoxia (2020). Turning vessels into vassals of cancer immunotolerance. Cancer Lett.

[142] De Palma M, Biziato D, Petrova TV (2017). Microenvironmental regulation of tumour angiogenesis. Nat Rev Cancer.

[143] Schaaf MB, Garg AD, Agostinis P (2018). Defining the role of the tumor vasculature in antitumor immunity and immunotherapy. Cell Death Dis.

[144] Lee HW, Seo HK (2021). Fibroblast growth factor inhibitors for treating locally Advanced/Metastatic bladder Urothelial Carcinomas via Dual Targeting of Tumor-Specific Oncogenic Signaling and the Tumor Immune Microenvironment. IJMS.

[145] Liu G, Chen T, Ding Z, Wang Y, Wei Y, Wei X (2021). Inhibition of FGF-FGFR and VEGF-VEGFR signalling in cancer treatment. Cell Prolif.

[146] Haibe Y, Kreidieh M, El Hajj H, Khalifeh I, Mukherji D, Temraz S (2020). Resistance Mechanisms to anti-angiogenic therapies in Cancer. Front Oncol.

[147] Gillis P, Savla U, Volpert OV, Jimenez B, Waters CM, Panos RJ (1999). Keratinocyte growth factor induces angiogenesis and protects endothelial barrier function. J Cell Sci.

[148] Pollard JW (2004). Tumour-educated macrophages promote tumour progression and metastasis. Nat Rev Cancer.

[149] Cross MJ, Claesson-Welsh L (2001). FGF and VEGF function in angiogenesis: signalling pathways, biological responses and therapeutic inhibition. Trends Pharmacol Sci.

[150] Taraboletti G, D’Ascenzo S, Borsotti P, Giavazzi R, Pavan A, Dolo V (2002). Shedding of the matrix metalloproteinases MMP-2, MMP-9, and MT1-MMP as membrane vesicle-associated components by endothelial cells. Am J Pathol.

[151] Tang T, Huang X, Zhang G, Hong Z, Bai X, Liang T (2021). Advantages of targeting the tumor immune microenvironment over blocking immune checkpoint in cancer immunotherapy. Sig Transduct Target Ther.

[152] Sackstein R, Schatton T, Barthel SR (2017). T-lymphocyte homing: an underappreciated yet critical hurdle for successful cancer immunotherapy. Lab Invest.

[153] Dirkx AEM, oude Egbrink MGA, Castermans K, van der Schaft DWJ, Thijssen VLJL, Dings RPM (2006). Anti-angiogenesis therapy can overcome endothelial cell anergy and promote leukocyte-endothelium interactions and infiltration in tumors. FASEB J.

[154] Oladipupo SS, Smith C, Santeford A, Park C, Sene A, Wiley LA (2014). Endothelial cell FGF signaling is required for injury response but not for vascular homeostasis. Proc Natl Acad Sci U S A.

[155] Murakami M, Nguyen LT, Zhuang ZW, Zhang ZW, Moodie KL, Carmeliet P (2008). The FGF system has a key role in regulating vascular integrity. J Clin Invest.

[156] Krysko DV, Garg AD, Kaczmarek A, Krysko O, Agostinis P, Vandenabeele P (2012). Immunogenic cell death and DAMPs in cancer therapy. Nat Rev Cancer.

[157] Kroemer G, Galluzzi L, Kepp O, Zitvogel L (2013). Immunogenic cell death in cancer therapy. Annu Rev Immunol.

[158] Fucikova J, Kepp O, Kasikova L, Petroni G, Yamazaki T, Liu P (2020). Detection of immunogenic cell death and its relevance for cancer therapy. Cell Death Dis.

[159] Hughes PE, Caenepeel S, Wu LC (2016). Targeted therapy and checkpoint immunotherapy combinations for the treatment of Cancer. Trends Immunol.

[160] Pfirschke C, Engblom C, Rickelt S, Cortez-Retamozo V, Garris C, Pucci F (2016). Immunogenic chemotherapy sensitizes tumors to checkpoint blockade therapy. Immunity.

[161] Garrido G, Rabasa A, Sánchez B, López MV, Blanco R, López A (2011). Induction of immunogenic apoptosis by blockade of epidermal growth factor receptor activation with a specific antibody. J Immunol.

[162] Shrimali RK, Yu Z, Theoret MR, Chinnasamy D, Restifo NP, Rosenberg SA (2010). Antiangiogenic agents can increase lymphocyte infiltration into tumor and enhance the effectiveness of adoptive immunotherapy of cancer. Cancer Res.

[163] Huang Y, Goel S, Duda DG, Fukumura D, Jain RK (2013). Vascular normalization as an emerging strategy to enhance cancer immunotherapy. Cancer Res.

[164] van E GDM. N H. A Window of Opportunity: Targeting Cancer Endothelium to Enhance Immunotherapy. Frontiers in immunology [Internet]. Front Immunol; 2020 [cited 2022 Jul 14];11. Available from: https://pubmed.ncbi.nlm.nih.gov/33262763/10.3389/fimmu.2020.584723 PMC7686513 33262763

[165] Fukumura D, Kloepper J, Amoozgar Z, Duda DG, Jain RK (2018). Enhancing cancer immunotherapy using antiangiogenics: opportunities and challenges. Nat Rev Clin Oncol.

[166] Schmittnaegel M, Rigamonti N, Kadioglu E, Cassará A, Wyser Rmili C, Kiialainen A (2017). Dual angiopoietin-2 and VEGFA inhibition elicits antitumor immunity that is enhanced by PD-1 checkpoint blockade. Sci Transl Med.

[167] Yamamoto Y, Matsui J, Matsushima T, Obaishi H, Miyazaki K, Nakamura K (2014). Lenvatinib, an angiogenesis inhibitor targeting VEGFR/FGFR, shows broad antitumor activity in human tumor xenograft models associated with microvessel density and pericyte coverage. Vasc Cell.

[168] Willett CG, Boucher Y, di Tomaso E, Duda DG, Munn LL, Tong RT (2004). Direct evidence that the VEGF-specific antibody bevacizumab has antivascular effects in human rectal cancer. Nat Med.

[169] Kelderman S, Schumacher TNM, Haanen JBAG (2014). Acquired and intrinsic resistance in cancer immunotherapy. Mol Oncol.

[170] Jenkins RW, Barbie DA, Flaherty KT (2018). Mechanisms of resistance to immune checkpoint inhibitors. Br J Cancer.

[171] Sharma P, Allison JP (2015). The future of immune checkpoint therapy. Science.

[172] Postow MA, Callahan MK, Wolchok JD (2015). Immune Checkpoint Blockade in Cancer Therapy. J Clin Oncol.

[173] Sharma P, Hu-Lieskovan S, Wargo JA, Ribas A (2017). Primary, adaptive, and Acquired Resistance to Cancer Immunotherapy. Cell.

[174] Petitprez F, Meylan M, de Reyniès A, Sautès-Fridman C, Fridman WH (2020). The Tumor Microenvironment in the response to Immune Checkpoint Blockade Therapies. Front Immunol.

[175] Binnewies M, Roberts EW, Kersten K, Chan V, Fearon DF, Merad M (2018). Understanding the tumor immune microenvironment (TIME) for effective therapy. Nat Med.

[176] Zhang Y, Chen L (2016). Classification of Advanced Human Cancers based on Tumor Immunity in the MicroEnvironment (TIME) for Cancer Immunotherapy. JAMA Oncol.

[177] Gettinger S, Politi K (2016). PD-1 Axis inhibitors in EGFR- and ALK-Driven Lung Cancer: Lost cause?. Clin Cancer Res.

[178] Rizvi NA, Hellmann MD, Snyder A, Kvistborg P, Makarov V, Havel JJ (2015). Cancer immunology. Mutational landscape determines sensitivity to PD-1 blockade in non-small cell lung cancer. Science.

[179] Cardarella S, Ogino A, Nishino M, Butaney M, Shen J, Lydon C (2013). Clinical, pathologic, and biologic features associated with BRAF mutations in non-small cell lung cancer. Clin Cancer Res.

[180] Cardarella S, Ortiz TM, Joshi VA, Butaney M, Jackman DM, Kwiatkowski DJ (2012). The introduction of systematic genomic testing for patients with non-small-cell lung cancer. J Thorac Oncol.

[181] Robins H, Desmarais C, Matthis J, Livingston R, Andriesen J, Reijonen H (2012). Ultra-sensitive detection of rare T cell clones. J Immunol Methods.

[182] Pylayeva-Gupta Y, Lee KE, Hajdu CH, Miller G, Bar-Sagi D (2012). Oncogenic Kras-induced GM-CSF production promotes the development of pancreatic neoplasia. Cancer Cell.

[183] Okuneye K, Bergman D, Bloodworth JC, Pearson AT, Sweis RF, Jackson TL. A validated mathematical model of FGFR3-mediated tumor growth reveals pathways to harness the benefits of combination targeted therapy and immunotherapy in bladder cancer. Comp Sys Onco [Internet]. 2021 [cited 2022 Apr 8];1. Available from: https://onlinelibrary.wiley.com/doi/10.1002/cso2.1019 10.1002/cso2.1019 PMC8722426 34984415

[184] Santiago-Walker AE, Chen F, Loriot Y, Siefker-Radtke AO, Sun L, Sundaram R (2019). Predictive value of fibroblast growth factor receptor (FGFR) mutations and gene fusions on anti-PD-(L)1 treatment outcomes in patients (pts) with advanced urothelial cancer (UC). JCO Wolters Kluwer.

[185] Benjamin DJ, Mar N, Rezazadeh Kalebasty A (2022). Immunotherapy with checkpoint inhibitors in FGFR-Altered Urothelial Carcinoma. Clin Med Insights Oncol.

[186] Rezazadeh A, Loriot Y, Papantoniou D, Siefker-Radtke AO, Necchi A, Naini V, et al. 757P an observational study of outcomes of patients (pts) with advanced urothelial carcinoma (UC) after anti-programmed death-(Ligand) 1 (PD-[L]1) therapy by fibroblast growth factor receptor gene alteration (FGFRa) status. Annals of Oncology. Volume 31. Elsevier; 2020. pp. 586–7.

[187] Cheng J, Li Y, Wang X, Dong Z, Chen Y, Zhang R (2021). Response stratification in the First-Line Combined Immunotherapy of Hepatocellular Carcinoma at genomic, transcriptional and Immune repertoire levels. J Hepatocell Carcinoma.

[188] Champiat S, Dercle L, Ammari S, Massard C, Hollebecque A, Postel-Vinay S (2017). Hyperprogressive Disease is a New Pattern of Progression in Cancer Patients treated by Anti-PD-1/PD-L1. Clin Cancer Res.

[189] Singavi AK, Menon S, Kilari D, Alqwasmi A, Ritch PS, Thomas JP (2017). Predictive biomarkers for hyper-progression (HP) in response to immune checkpoint inhibitors (ICI) – analysis of somatic alterations (SAs). Ann Oncol.

[190] Li G, Choi JE, Kryczek I, Sun Y, Liao P, Li S (2023). Intersection of immune and oncometabolic pathways drives cancer hyperprogression during immunotherapy. Cancer Cell.

[191] Kursunel MA, Esendagli G (2016). The untold story of IFN-γ in cancer biology. Cytokine Growth Factor Rev.

[192] Forys JT, Kuzmicki CE, Saporita AJ, Winkeler CL, Maggi LB, Weber JD (2014). ARF and p53 coordinate tumor suppression of an oncogenic IFN-β-STAT1-ISG15 signaling axis. Cell Rep.

[193] Zhang W, Xia H, Yang R, Zhang Y, Zheng Q, Shang X (2022). Fibroblast growth factor receptor family mutations as a predictive biomarker for immune checkpoint inhibitors and its correlation with tumor immune microenvironment in melanoma. Front Immunol.

[194] W GGLCFZTJ et al. W, S M,. Special issue “The advance of solid tumor research in China”: FGFR4 alterations predict efficacy of immune checkpoint inhibitors in nonsmall cell lung cancer. International journal of cancer [Internet]. Int J Cancer; 2023 [cited 2023 Jan 29];152. Available from: https://pubmed.ncbi.nlm.nih.gov/36062503/10.1002/ijc.34239 36062503

[195] Necchi A, Raggi D, Giannatempo P, Marandino L, Farè E, Gallina A (2021). Can patients with muscle-invasive bladder Cancer and fibroblast growth factor Receptor-3 alterations still be considered for Neoadjuvant Pembrolizumab? A Comprehensive Assessment from the updated results of the PURE-01 study. Eur Urol Oncol.

[196] Sj SM, K TDNAC et al. Y, Y W,. TGFβ attenuates tumour response to PD-L1 blockade by contributing to exclusion of T cells. Nature [Internet]. Nature; 2018 [cited 2022 Apr 13];554. Available from: https://pubmed.ncbi.nlm.nih.gov/29443960/10.1038/nature25501 PMC6028240 29443960

[197] Uehara Y, Watanabe K, Hakozaki T, Yomota M, Hosomi Y (2022). Efficacy of first-line immune checkpoint inhibitors in patients with advanced NSCLC with KRAS, MET, FGFR, RET, BRAF, and HER2 alterations. Thorac Cancer.

[198] Noguchi T, Ward JP, Gubin MM, Arthur CD, Lee SH, Hundal J (2017). Temporally distinct PD-L1 expression by tumor and host cells contributes to Immune escape. Cancer Immunol Res.

[199] O’Donnell JS, Long GV, Scolyer RA, Teng MWL, Smyth MJ (2017). Resistance to PD1/PDL1 checkpoint inhibition. Cancer Treat Rev.

[200] Gutierrez M, Subbiah V, Nemunaitis JJ, Mettu NB, Papadopoulos KP, Barve MA (2020). Safety and efficacy of pemigatinib plus pembrolizumab combination therapy in patients (pts) with advanced malignancies: results from FIGHT-101, an open-label phase I/II study. JCO Wolters Kluwer.

[201] Siefker-Radtke AO, Currie G, Abella E, Vaena DA, Rezazadeh Kalebasty A, Curigliano G (2019). FIERCE-22: clinical activity of vofatamab (V) a FGFR3 selective inhibitor in combination with pembrolizumab (P) in WT metastatic urothelial carcinoma, preliminary analysis. JCO Wolters Kluwer.

[202] Subbiah V, Iannotti NO, Gutierrez M, Smith DC, Féliz L, Lihou CF (2022). FIGHT-101, a first-in-human study of potent and selective FGFR 1–3 inhibitor pemigatinib in pan-cancer patients with FGF/FGFR alterations and advanced malignancies. Ann Oncol.

[203] Kacew A, Sweis RF (2020). FGFR3 alterations in the era of immunotherapy for urothelial bladder Cancer. Front Immunol.

[204] Wu T, Dai Y (2017). Tumor microenvironment and therapeutic response. Cancer Lett.

